# Molecular dynamics of the histamine H3 membrane receptor reveals different mechanisms of GPCR signal transduction

**DOI:** 10.1038/s41598-020-73483-5

**Published:** 2020-10-09

**Authors:** Leonardo David Herrera-Zúñiga, Liliana Marisol Moreno-Vargas, Luck Ballaud, José Correa-Basurto, Diego Prada-Gracia, David Pastré, Patrick A. Curmi, Jean Michel Arrang, Rachid C. Maroun

**Affiliations:** 1 UMR-S U1204, Structure et Activité de Biomolécules Normales et Pathologiques, INSERM/Université d’Evry-Val d’Essonne/Université Paris-Saclay, 91000 Evry, France; 2 Computational Biology and Drug Design Research Unit, Federico Gómez Children’s Hospital of Mexico City, Mexico City, Mexico; 3 Laboratoire de Neurobiologie et Pharmacologie Moléculaire, INSERM U894, Centre de Psychiatrie et Neurosciences, 75014 Paris, France; 4 Área de Estudios de Posgrado e Investigación, Tecnológico de Estudios Superiores del Oriente del Estado de México, Los Reyes Acaquilpan, Mexico; 5 Laboratorio de Modelado Molecular y Bioinformática, Escuela Superior de Medicina, Instituto Politécnico Nacional, Mexico City, Mexico

**Keywords:** Molecular neuroscience, Computational models, Protein structure predictions, Molecular modelling

## Abstract

In this work, we studied the mechanisms of classical activation and inactivation of signal transduction by the histamine H3 receptor, a 7-helix transmembrane bundle G-Protein Coupled Receptor through long-time-scale atomistic molecular dynamics simulations of the receptor embedded in a hydrated double layer of dipalmitoyl phosphatidyl choline, a zwitterionic polysaturated ordered lipid. Three systems were prepared: the apo receptor, representing the constitutively active receptor; and two holo-receptors—the receptor coupled to the antagonist/inverse agonist ciproxifan, representing the inactive state of the receptor, and the receptor coupled to the endogenous agonist histamine and representing the active state of the receptor. An extensive analysis of the simulation showed that the three states of H3R present significant structural and dynamical differences as well as a complex behavior given that the measured properties interact in multiple and interdependent ways. In addition, the simulations described an unexpected escape of histamine from the orthosteric binding site, in agreement with the experimental modest affinities and rapid off-rates of agonists.

## Introduction

G-protein coupled receptors (GPCR) constitute the largest family of integral-membrane signaling receptors and their topology consists of a bundle of seven transmembrane α-helices (TM1–TM7). The N-terminus (N-ter) is extracellular. Because the number of TM helices is uneven, the C-terminus (C-ter) is intracellular. Connecting the helices are three extracellular loops (EC1–ECL3) and three intracellular loops (ICL1–ICL3). GPCRs belong to the superfamily of 7TM receptors^1–3^. The histamine receptors (H1R, H2R, H3R and H4R) are members of the biogenic amine receptor subfamily of GPCRs and present, in addition to the TMs, a short C-ter amphipathic juxta-membrane helix 8 (H8) on the cytoplasmic side, oriented parallel to the membrane, making contacts with this one and inserting a Cys-palmitoyl modification in it. The rat H3R (rH3R) was originally discovered in the brain on histaminergic neurons as a presynaptic auto-receptor and heteroreceptor inhibiting the synthesis and the depolarization-induced release of histamine (HSM; Supplementary Fig. S1a)^4^. H3R is predominantly expressed in the central nervous system (CNS) and to a lesser extent in the peripheral nervous system^5^. The histamine receptors’ interaction with HSM, the endogenous neurotransmitter agonist, elicits a variety of physiological effects, including allergic reactions (H1R)^6^, gastric acid secretion (H2R)^7^, mediation of neurotransmitter release and the inhibition of cAMP production (H3R)^4^, and immunological response (H4R)^8^. HSM is synthetized and released by histaminergic neurons. It plays a major role in cognition and other physiological functions such as vigilance, attention, impulsivity and feeding/weight regulation^9^. It is stocked in vesicles and released after an electrical stimulus. HSM binds pre- or post-synaptic receptors. Ciproxifan^4,10,11^ (CPX or FUB-359; CAS No. 184025-18-1; GRAC database, guidetopharmacology.org; Supplementary Fig. S1b) is a highly potent and selective competitive H3-receptor antagonist/inverse agonist with pro-cognitive properties and a nanomolar affinity (for a given signaling assay used, inverse agonism refers to the ability of a compound to inhibit constitutive GPCR signaling, presenting thus negative efficacy)^12^.

Cloning of the histamine H3R cDNA in 1999 allowed detailed studies of its molecular aspects and indicated that H3R can activate several signal transduction pathways^4^. H3R is regarded as a potential therapeutic target because of its location in the CNS and for the modulation of a variety of functions such as cognitive processes, epilepsy, food intake and sleep-wakefulness^13,14^. The transmembrane region of H3R is often the site of ligand and drug interaction. Several H3R antagonists/inverse agonists appear to be promising drug candidates^15–17^. Three-dimensional (3D) atomistic models of antagonist–receptor complexes have been used to investigate the details of ligand and drug interactions with H3R and have been successful in providing important insights regarding their binding; additionally, several groups have reported the features of the general H3R pharmacophore. This approach has been particularly successful for investigating GPCR/ligand binding modes and is complementary to 3D receptor/ligand modeling. The features of this antagonist pharmacophore are a primary basic group, either a piperidine or pyrrolidine, which is connected by an alkyl linkage to a second group. Other groups have observed that the addition of a second basic group increases the binding affinity^18^. Across the superfamily of GPCRs, there exist many residues that have been conserved throughout evolution and are thus thought to play key roles in receptor structure and/or function. Site-directed mutagenesis has demonstrated the importance of many of these residues in several different biogenic amine receptors, including some of the histamine receptors^19^ (and references therein). Human H3R is sensitive to monovalent cations such as sodium^20,21^. The interaction of the cation with Asp 2.50 facilitates binding to residues in other TM domains. The critical role of TM5 has been demonstrated in many receptors (β2-adrenergic, H1, H2, etc.), including the H3R^22^. On another hand, the existence of distinct active and inactive conformations of H3R has been established in vitro and in vivo via the pharmacological concept of protean agonism^23,24^.

In the absence of the experimental structure of H3R, several computational studies have been carried out for determination of the binding of several antagonists to H3R using a homology model for the receptor and employing the continuum dielectric approximation for the surrounding bilayer environment. Thus, based on the crystals of rhodopsin^25^, 3D in silico models for H3R have been obtained in the past^4,12,18,22,26–32^. Nevertheless, these models suffer from a variety of imperfections during their building, such as the use of incorrect or absent alignments; manual adjustments and manipulations; energy refinement in vacuo; short molecular dynamics (MD) trajectories (1–10 ns); and lack of consideration of the state of the template structure (active/inactive). Other models do not contain any ligands^33^. In other instances, modeling of GPCRs only pretended to represent TM domains of receptor structures aimed at studying receptor-antagonist interactions and not their activated states^34^.

In this work, we generated curated in silico 3D structural models of H3R in the active, inactive and constitutive states, this latter being represented by a ligand-devoid receptor in an active state. As MD simulations provide a unique opportunity for studying molecular mechanisms, our approach consisted in simulating the binding of known H3 ligand compounds to the receptor model to study and analyze the spatiotemporal behavior of the resulting H3R–ligand complexes through trajectories totaling more than 3 µs. We thus proceeded to embed the receptor models in a hydrated, ionized and electrically neutral DPPC phospholipid bilayer. An extensive analysis of the results of the trajectories indicated that each state of the receptor is described by many different structural and dynamical properties. These properties are interdependent, showing an intricate network of short- and long-distance crosstalk between distinct regions of the receptor, a fingerprint of complex behavior. In addition, the MD simulations showed a spontaneous escape of HSM from the orthosteric binding site, preceded by a short binding step in the extracellular vestibule during the unbinding pathway.

## Results

### Free energy landscapes suggest a multiplicity of conformations for each state of the receptor and between receptor states

We obtained the initial structures for the two holo-systems, that is, the HSM–H3R complex and the CPX–H3R complex through ligand docking to the orthosteric site of the receptor.

**Figure 1:**
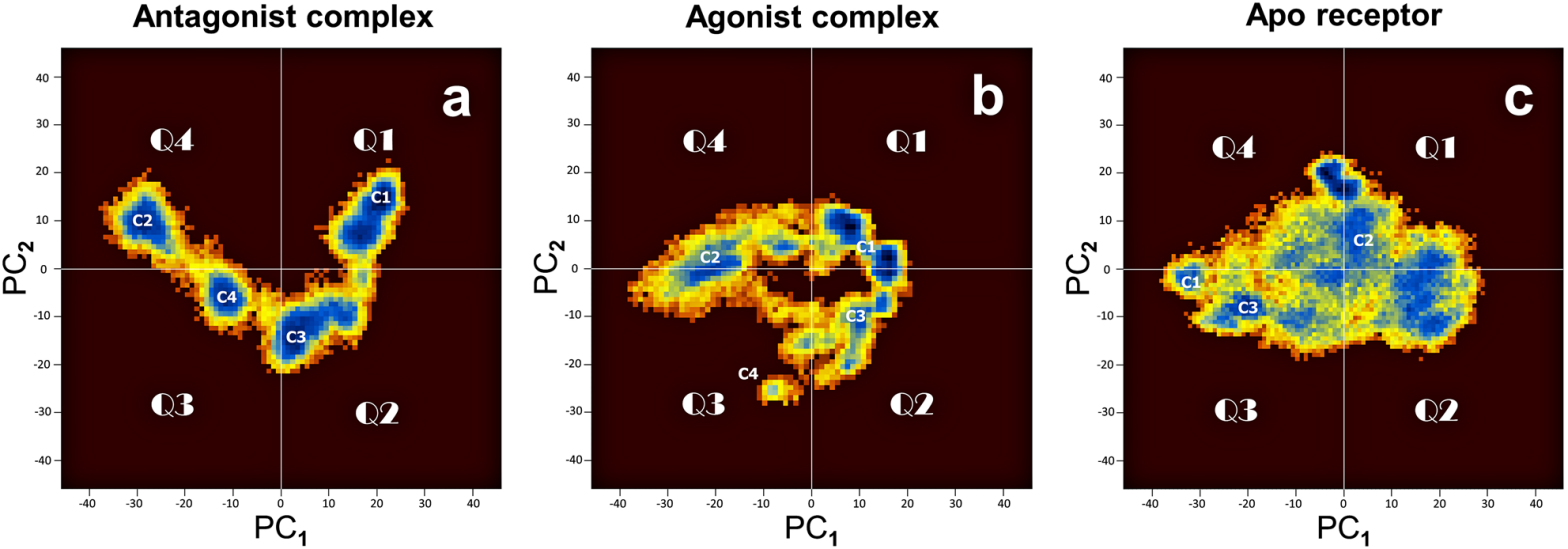
Free energy landscape. PCA-based free energy landscape of the structures of the three systems identified by cartesian Principal Component Analysis. The panels (**a**–**c**) show a pseudo color representation of the distribution of the first two principal components PC1, PC2 obtained from a ∼ 1 µs MD simulation for the antagonist, agonist and apo structures, respectively. Blue color represents energy wells (C1, C2, etc.). The map is divided into four quadrants (Q1–Q4).

Figure 1a shows the two-major component PC1–PC2 2D plot of the cartesian Principal Component Analysis (PCA) obtained for the protein structure of the *antagonist–H3R* complex. In the presence of CPX, the plot shows a V-shaped energy landscape with four populations: clusters C1, C2, C3 and C4, located in quadrants 1 to 4 (Q1–Q4). Figure 1b shows the PC1–PC2 plot for the structure of H3R in the *agonist–H3R* complex when the agonist was still bound to H3R. It is composed of three major clusters—C1, C2 and C3—and a minor one (C4 in Q3). The PC1–PC2 plot for the *apo* receptor is in Fig. 1c. The occupied region in the map is rather large and diffuse, presenting several energy clusters with a marked cluster at the Q1/Q4 border (C2).

The evidence of constitutive activity of the apo receptor can be seen when comparing energy landscapes of Fig. 1b and 1c in which we observe in both a diffusiveness and a shape resemblance, with the landscape of the agonist complex being a subset of that of the apo receptor. These features point at an apo receptor showing basal activity, although the nature of this active state is different from that of the agonist-bound receptor.

The PCA maps for the receptor show thus distinct morphologies, and number and depths of the conformation populations, reflecting the existing conformational heterogeneity for each state. Each receptor has a major native/representative structure dictated by the type of ligand bound to it. However, the 3D structure spaces may overlap, leading to confluence regions in which any one system may present at one time or another the structural characteristics of the other system. These overlapping regions suggest that, within the intrinsic structural fluctuations specific to each state, each state may visit at one time or another, an alternative state. Finally, the PCA clusters are structurally consistent with the metastable conformational states evidenced through the RMSD matrices (SI).

### Agonist and inverse aFree energy landscapes suggestgonist establish differential interactions with the receptor

**Figure 2:**
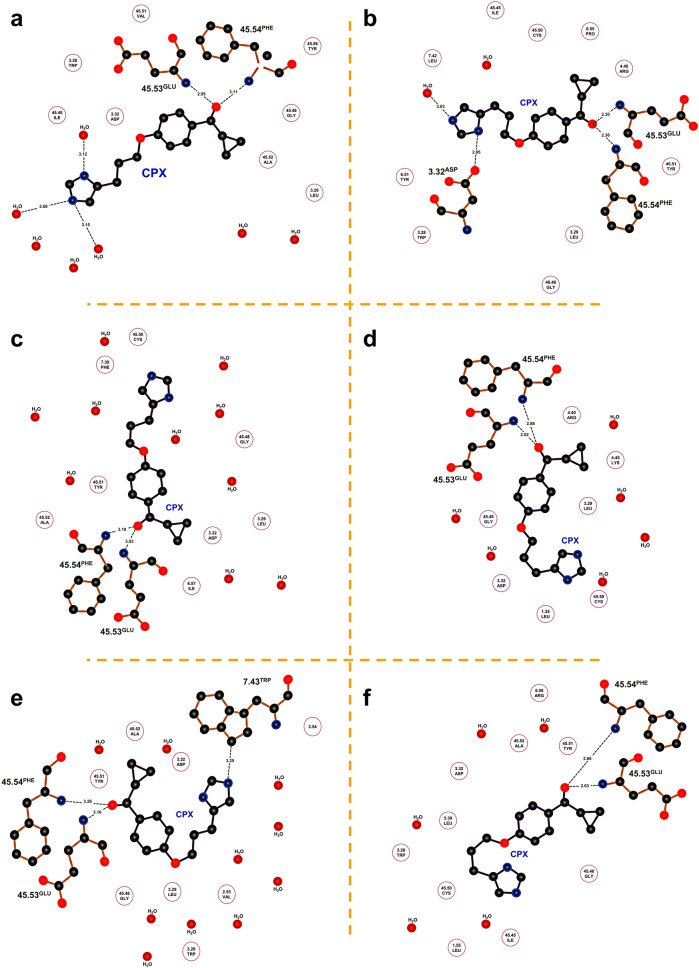
Ciproxifan binding modes. 2D-binding mode of ciproxifan within the H3 receptor. Polar interactions and H-bonds (green lines) between the amino acids (in circles), water (red dots) and ciproxifan ligand (CPX) can be appreciated. The (**a**–**f**) panels show the most relevant configurations and bioconformations of CPX throughout the simulation. For torsion angles ϕ_1_–ϕ_4_, the bioconformations include: (**a**) an extended or all-*trans* chain; (**b**) a “zig-zag” chain; (**c**) a “paddle” chain; (**d**) a “cyclic” conformation that approaches Cδ2 of the imidazole with C11 (the carbon bound to the ether oxygen), forming a virtual 5-membered ring; (**e**, **f**) two “closed” or *cis* conformations. In the last frame of the trajectory, CPX adopts the conformation in (**a**). The dihedral connecting the carbonyl oxygen and the cyclopropane, ϕ_5_, adopts two states: perpendicular (− 90° to − 110°) or co-planar (± 180°) to the aromatic ring.

For CPX, the carbonyl oxygen between the cyclopropane and the phenoxy ring (Supplementary Fig. S1b) is in a long-lasting (> 70% of residence time) bifurcated bond with the N atoms of the main chains of Glu 45.53 and Phe 45.54 (ECL2) (Fig. 2a). Instead, the latter interacts at one time or another with either the internal solvent molecules or different neighboring residues because of the mobility that single bonds give to the imidazole ring; none of these interactions is significantly populated. As shown in the LigPlot + diagram^35^ of Fig. 2a, the imidazole moiety is associated with three water molecules. In Fig. 2b, this moiety is H-bonded to Asp 3.32 and a water molecule, whereas in Fig. 2c–f, the imidazole ring shows no H-bonds to water molecules. In Fig. 2e, the imidazole is H-bonded to the N3 of the indole of Trp 7.43. Other contacts along the trajectory are listed in Table 1 and include all residues within 4 Å of the CPX ligand with large residence times. Residues with 70% or more in contact with CPX include three Cys, two Phe and one of each Leu, Ala, Val, Tyr, Trp, Asp, Glu, and Gly. Note the presence of the two acidic residues Asp and Glu.

**Table 1:** Interactions between the ciproxifan antagonist/inverse agonist and the receptor for residence times greater than 70%.

H3R ANTA		
Residue		Residence time (%)
PHE192	45.54	100.00
CYS188	45.5	100.00
ALA190	45.52	100.00
LEU111	3.29	100.00
TYR189	45.51	99.00
TRP110	3.28	99.00
ASP114	3.32	99.00
CYS87	2.57	97.00
GLY186	45.48	96.00
GLU191	45.53	96.00
CYS107	3.25	93.00
VAL83	2.53	83.00
PHE280	7.39	77.00

**Figure 3:**
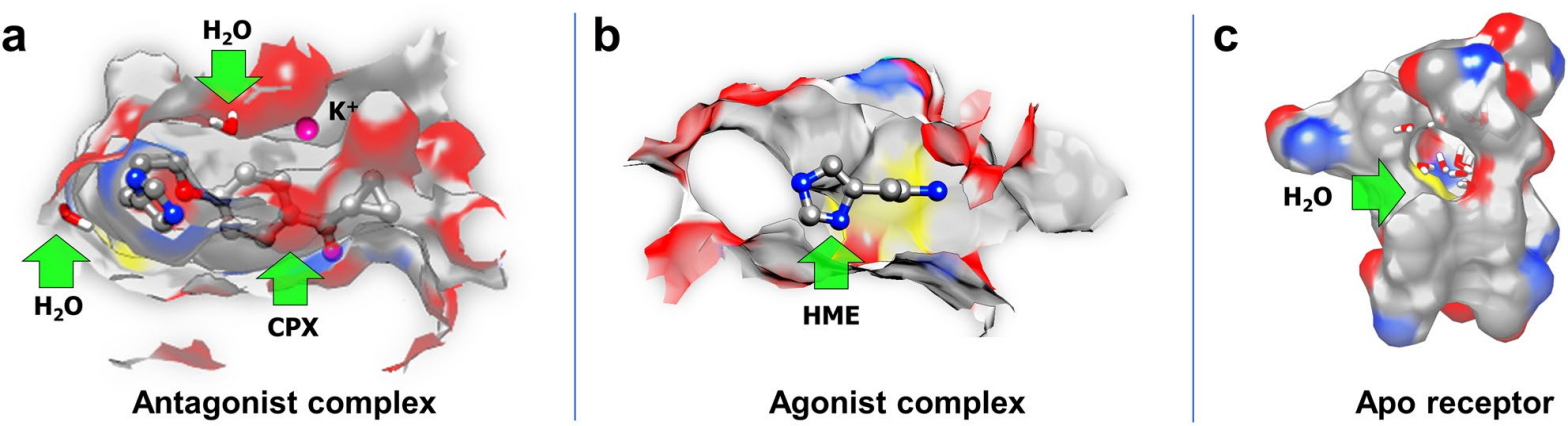
Receptor binding pocket. Side view of the H3R ligand binding pocket showing binding modes for (**a**) the antagonist complex, (**b**) the agonist complex and (**c**) the apo receptor (devoid of ligand), respectively, as resulting from an optimal structural alignment of the TM helices of each system. The average number of water molecules in the orthosteric site is of 61, 35, and 49, respectively. Binding pose f (Fig. 2f) is shown in (**a**), as well as the K^+^ ion.

Thus, the LigPlot + plots in Fig. 2 show the flexibility of the imidazole-containing moiety of CPX, reflected in the wide range of values the different single-bond torsion angles adopted during the MD trajectory (t, g+, g−, − 120°), leading to essentially six bioactive conformations (a–f). Figure 3a shows a CPX binding mode.

**Figure 4:**
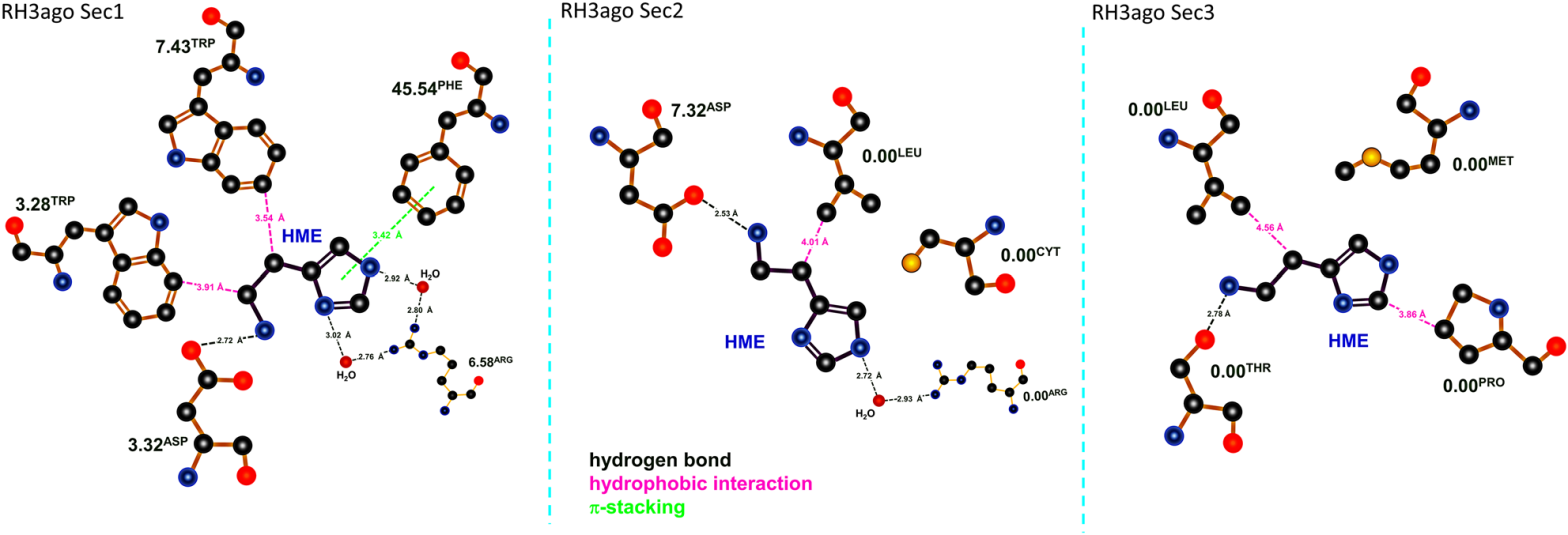
Histamine binding modes. 2D-binding mode of histamine within the H3 receptor. Polar interactions and H-bonds between the amino acids, water and histamine ligand (green lines); hydrophobic interactions (magenta lines); and π-stacking (light green lines) can be appreciated. The three panels correspond to sections 1–3 in the trajectory and show the most relevant configurations and conformations of histamine throughout the simulation.

For HSM, the number of residues most in contact are Asp 3.32, Trp 3.28 and Trp7.43, and Phe 45.54 (ECL2) (Table 2; Fig. 4, corresponding to section 1 of the trajectory). Figure 3b shows an HSM binding mode corresponding to section 1.

**Table 2:** Interactions between the histamine agonist and the receptor for the first period of the production trajectory, when the agonist is fully bound to the receptor.

HR3 AGO		
SECTION 1		
Residue		Residence time (%)
ASP114	3.32	97.20
TRP110	3.28	94.60
TRP291	7.43	88.10
PHE192	45.54	76.90

Residence times greater than 70%.

The binding pocket of the *apo* receptor, along with internal water molecules, is in Fig. 3c.

### K^+^-dependent conformational changes: diffusion into the sodium allosteric site of the antagonist complex

During the production run, a potassium monocation from the bulk solvent spontaneously diffused into the sodium allosteric pocket of the antagonist–H3R complex, near Asp2.50. The diffusion of the cation is extremely fast (~ 100 fs; Supplementary Fig. S19, yellow line). It is hydrated by five water molecules in the aqueous milieu, three during its path to the interior of the receptor, and five in the final stage, when in the Na^+^ allosteric site. The presence of K^+^ in the antagonist–H3R complex argues for a role equivalent to that of Na^+^ in the stabilization of the inactive state^36^. In addition, K^+^ reduced H3R-regulated signaling in the same way as Na^+^^20^. Thus, no significant energy barriers in K^+^ ion permeation appear in the path from the cytosol to the allosteric binding site in the lower part of the TM domain through an opening of the hydrated space within the receptor. This suggests that this transition locks the receptor in the inactive state conformation. By analogy, we assume that Na^+^ follows a similar entrance pathway to K^+^.

Finally, Arrang and co-workers^22^ observed that the presence of Ca^2+^ down-regulated the H3R receptor. We attribute this effect to an allosteric binding site for Ca^2+^ at the extracytoplasmic region and/or at the sodium binding site (their ionic radius − 114 and 116 pm, respectively- are very similar).

### CPX forms a stable complex in the orthosteric site; HSM shows several binding modes and eventually vacates the binding site spontaneously

We found that there were multiple non-covalently bound states of HSM corresponding to the activated state of H3R due to the small size of the agonist and its ability to establish stabilizing non-bonded interactions in distinctly different binding orientations with surrounding residues. Indeed, during the docking simulation, HSM showed several major binding modes in several binding subpockets of the orthosteric site. For the starting structure, we chose the one compatible with experimental results with H2R in which the quaternary N_ζ_ of HSM interacts with Asp 3.32 (Asp98 of H2R; Asp114 of H3R), highly conserved through class A GPCRs and essential for HSM binding and action, serving as a counter-anion to the cationic amine moiety of HSM, and agonist and antagonist binding^37,38^. The multiple poses we found are compatible with the experimental findings of Gantz et al.^37^ in which removal of the negatively charged amino acid abolished HSM-stimulated increases in cellular cAMP, but not HSM binding to the receptor. Our results support thus HSM’s multiple binding poses and suggest that the pose leading to interaction with Asp3.32 leads to activation of the receptor. For CPX, we chose a binding pose in which the imidazole moiety is in contact with Asp 3.32. The binding pockets of the two ligands are thus partially shared. There may be a secondary allosteric low-affinity binding site for agonists, as seen by binding kinetics^4,22^, but in this study we focus on the orthosteric site only.

**Figure 5:**
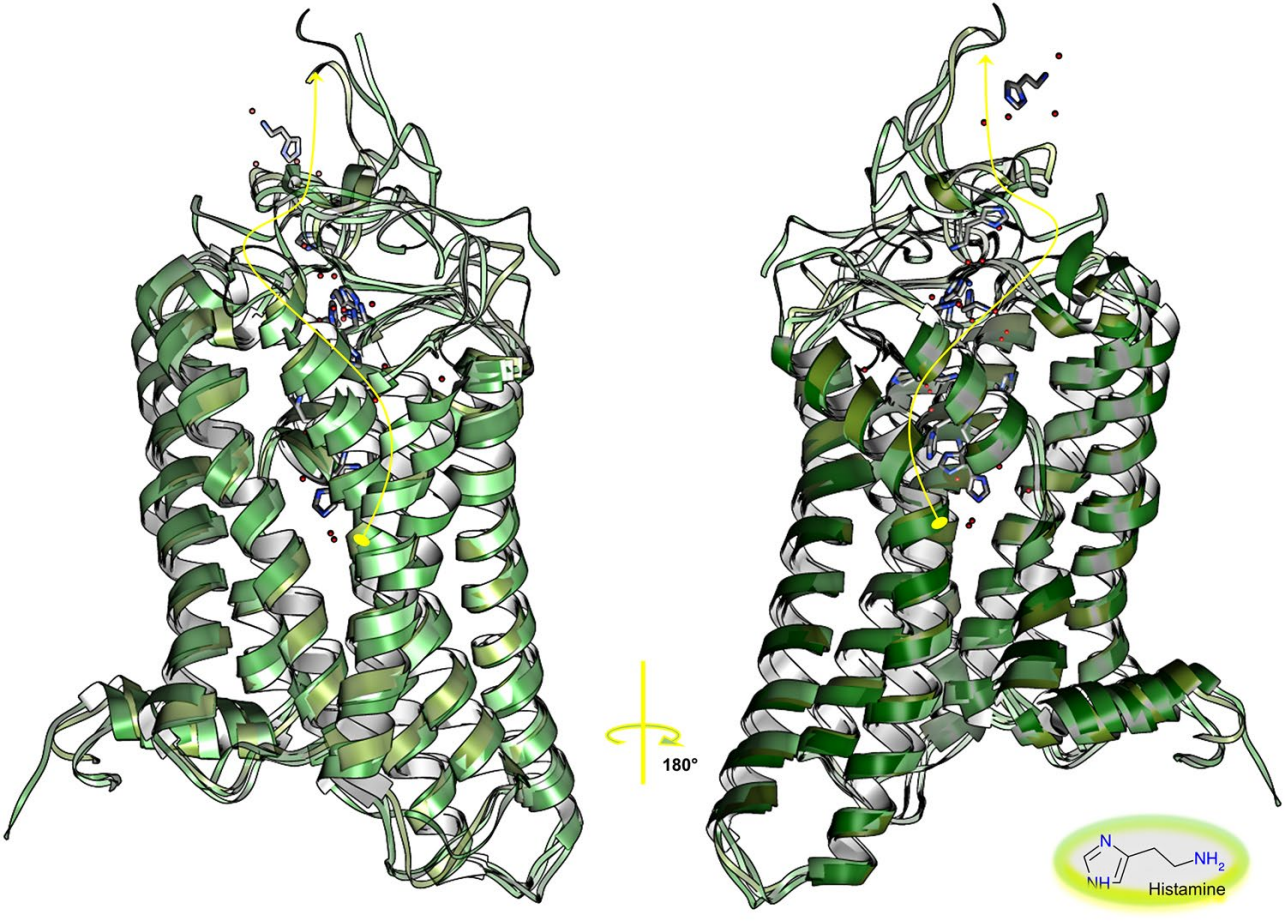
Histamine unbinding pathway. Schematic illustration of the four phases of the spontaneous unbinding and exit pathway of the HSM ligand (curve in yellow), showing the pathway from its lodging in the binding cavity (section 1), with interactions of either the imidazol or the amino group with TM2, TM3, ECL2, TM6 and TM7; to its disposal from it (section 2) with interactions with N-ter, TM3, ECL2, TM6 and TM7; to its small residence time in the vestibular area formed by N-ter, TM3, ECL2 and TM7 (section 3); and to its final escape into the solvent (section 4). Several of the previous interactions are mediated by water.

Interestingly, during the MD simulation, the agonist–H3R complex showed an unexpected behavior in which the HSM ligand, albeit at given times deep in the binding cleft, eventually escaped spontaneously from the binding site. We thus defined four trajectory time intervals to better describe the unbinding process. The first interval (section 1) during which the ligand is bound to the orthosteric binding cavity, comprises from nanosecond 112 of the MD production phase, when the RMSD of Cα atoms attains a plateau, to nanosecond 590. In this interval, the HSM displays, nevertheless, a very high mobility despite the solvent molecules filling the cleft. The N-ter folds over ECL2 and results in a salt bridge between Arg27 (N-ter) and Glu 45.53 (ECL2), with ECL2 folding over the cleft, capping the binding pocket. The second interval (section 2), a transition, includes the beginning of the ligand out of the site but still bound to the transmembrane part of the receptor, lasting 1.15 ns. The N-ter then begins to unfold and ECL2 moves aside following the disruption of the salt bridge. The third interval (section 3) comprises the positively charged ligand trapped between the extramembrane N-ter and ECL2 regions and the anionic heads of the upper leaflet of the phospholipids; it lasts almost 1 ns. Thus, it involves the extracellular vestibule, but it does not seem to represent a metastable binding site^39^. The binding cleft is then open to the extracellular space. The fourth and last interval (section 4; 592 ns–1.02 µs) in the unbinding pathway defines the exit of the ligand to join the bulk of the solvent. The N-ter now forms the lid of the cleft, with ECL2 remaining “aside.” In the bulk, HSM shows a hydration shell of about 30 water molecules and interacts eventually with a chloride ion through its alkyl amine. HSM binds from time to time to the extracellular vestibule. The exit pathway of HSM is illustrated in Fig. 5 (yellow line) and involves N-ter, TM3, ECL2, TM6 and TM7 for section 2; N-ter, TM3, ECL2 and TM7 for section 3; and the solvent for section 4. The HSM exit pathway (see below) and K^+^ entrance pathways were different (Fig. 5 vs. Supplementary Fig. S19).

### Water molecules in the internal cavity

At constant number, the water molecules are very mobile in the orthosteric cavity. In addition, their number depends on the state of the receptor, and, of course, on the size and flexibility of the ligand bound to it. Supplementary Table S2a shows the average water population in the cavity (61, 35 and 49 for the antagonist complex, the agonist complex, and the apo receptor, respectively).

Several of these water molecules mediate H-bonds between the receptor and the ligands and play important roles not only in the activation processes, but also in inactivation and spontaneous activation (Supplementary Fig. S11).

### Diffusion of a potassium monocation into the antagonist complex sodium allosteric site

This study also shows that the monovalent K^+^ spontaneously penetrates the antagonist-bound receptor to bind the Na^+^ allosteric site, in analogy to the D2 receptor (de Fabritiis and coll); it can thus be an endogenous allosteric modulator enhancing antagonist binding.

### Specific lipid-binding sites on the receptor

We observed that the lipid-binding sites in the receptor depend on the state of the receptor (SI). In other words, the location, number and structure of the lipid-binding site will recognize the state of the receptor. Meaningfully, the lipids bind to specific TM helices and are localized in different regions (upper and lower leaflets) of the bilayer (Supplementary Figs. S12–S14; Supplementary Tables S4–S6). Our analyses also highlight the presence of distinctly different lipid allosteric regulatory sites according to the state of the receptor^40^ and point to the mechanism of modulation of lipid on receptor function^22,41,42^—each specific protein-lipid interaction stabilizes the corresponding state of the receptor (Supplementary Fig. S12). Therefore, like cholesterol binding to β2-AR, there are specific binding sites for DPPC in H3R, but specific to the state of the receptor. Like palmitic acid and cholesterol in the β2-AR-carazolol crystal structure (PDB code 2RH1), the contacts between the lipids’ hydrocarbon chains and hydrophobic receptor residues follow the hydrophobic matching principle. However, as we found, the specific lipid-binding motifs on the membrane protein surface consist also of positively charged residues that specifically interact with the negatively charged phosphodiester groups (Supplementary Figs. S12–S14). Our findings are thus consistent with the experimentally reported lipid binding sites in membrane proteins^22,43^, and with the protein-head and protein-tail contacts for the H1R (PDB ID 3RZE), as reported in the MemProtMD database^22^. From the analysis of the amino acid residues in contact with lipids for each of the three systems, we can formulate lipid binding sequence motifs (Supplementary Table S8).

## Discussion and Conclusions

GPCR dynamics are important from a structural and functional viewpoint, including conformational changes and rearrangements of the transmembrane helices^44^. In this work, we have explored with atomistic MD simulations many microscopic spatial and temporal properties of the classical activation and inactivation pathways of the second step (see below) of signal transduction pre-activation by the H3 receptor that are not easily accessible by experiment, including new undocumented interactions. Of course, other G-protein independent activation mechanisms exist^45^S2.

The average values of several indicative properties, such as the RMSD and the RMSD matrix (Supplementary Figs. S2, S3), remain roughly constant with increased sampling, even though the production run temperature was relatively high (323.15 K), leading us to believe that the 1 µs MD simulations for each of the three systems sample satisfactorily the phase space^46^. Furthermore, we ran two replica small trajectories that pointed to the general character of our observations. An apparent paradox is that we can observe significant phenomena during MD simulations that span 1 µs, whereas the time constant for the activation switch of a GPCR can be in the tens to hundreds of ms. For example, for α2A-AR in living cells the rate constant was < 40 ms^47^; these authors also refer to other results that indicate that the entire GPCR-signaling chain can be activated within 200–500 ms. The time-scale difference between theory and experiment is due, in our view, to the fact that we have studied only a confined stage of the entire GPCR signaling cascade whose individual elements comprise at least (1) ligand binding, (2) conformational change of the receptor (preliminary activation or inactivation), (3) interaction between the ligand–complex receptor and the G-protein (full activation), (4) G-protein conformational changes including GDP release and GTP binding, (5) G protein–effector interaction, (6) change in effector activity, and (7) the resulting ion conductance or second messenger concentration changes^48^. Steps 1–2 are dovetails with the ternary complex model for GPCRs^49,50^ and correspond to the Ri → ARi → ARa schemes of the extended or cubic complex models (Ri, initial state unbound receptor; ARi, ligand-complexed receptor in the initial state; ARa, ligand-complexed receptor in the active/inactive state). We may add receptor oligomerization between steps 2 and 3 when applicable. But our simulations do not deal with the whole activation process, rather with step 2 only—the initial structures represent already the docked complexes—and can be considered to span it well, even though the H3R in any of the two ligand-bound described states is not in a fully active or inactive state, rather in a “pre-active” state since it is not bound to its G-protein (Gi/o) to form the ternary complex, but in which the receptor is able to interact with it^51,52^.

**Figure 6:**
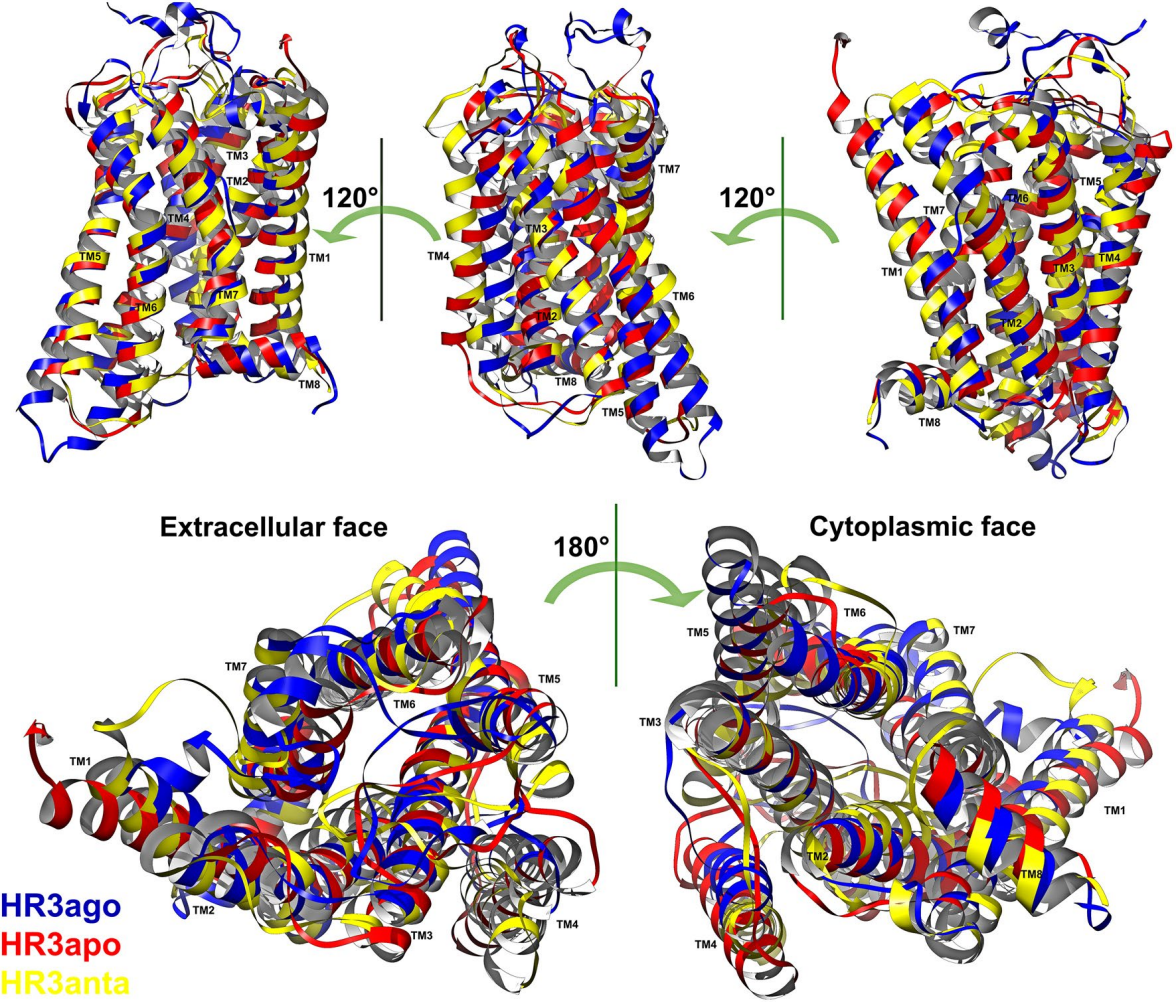
Representative 3D structures. 3D superposition of the production-phase average representative structure of each system. In yellow, the antagonist-H3R complex; in blue the agonist-H3R complex; in red the apo receptor. In the extracellular view, the next exposed TM helix for apo H3R is TM5, whereas the inner most TM helix is TM5 for the antagonist complex.

An illustration of the conformational fluctuations of the receptor due to ligand binding, i.e. pre-activation, is the agonist-induced conformational changes of β2-AR, which include a large outward movement at the cytoplasmic end of TM6 and an α-helical extension of the cytoplasmic end of TM5^53,54^. This is also observed in the collective motions of the TM5, TM6 and TM7 helices of H3R (Fig. 6), supporting the view that several receptor collective movements are common throughout class A GPCRs. Incidentally, the peripheral TM4 is more eccentric in the order antagonist complex > apo receptor > agonist complex.

Kong and Karplus^55^ studied the signal transduction mechanism of rhodopsin by MD simulations of the high resolution, inactive structure in an explicit membrane environment. Even though we are dealing with H3R and not rhodopsin, and that the ligands are not the same, several of our results are analogous, indicating shared structural features in the general mechanism of class A GPCR activation^56^. Thus, we observe correlated movements of TM6 and TM7 around the class A GPCR highly conserved Pro 6.50, and Pro 7.50 of the CWXP and NPXXY motifs. In addition, Kong and Karplus found that the major signal-transduction pathway involves the interdigitating side chains of helices VI and VII, just like we do.

**Figure 7:**
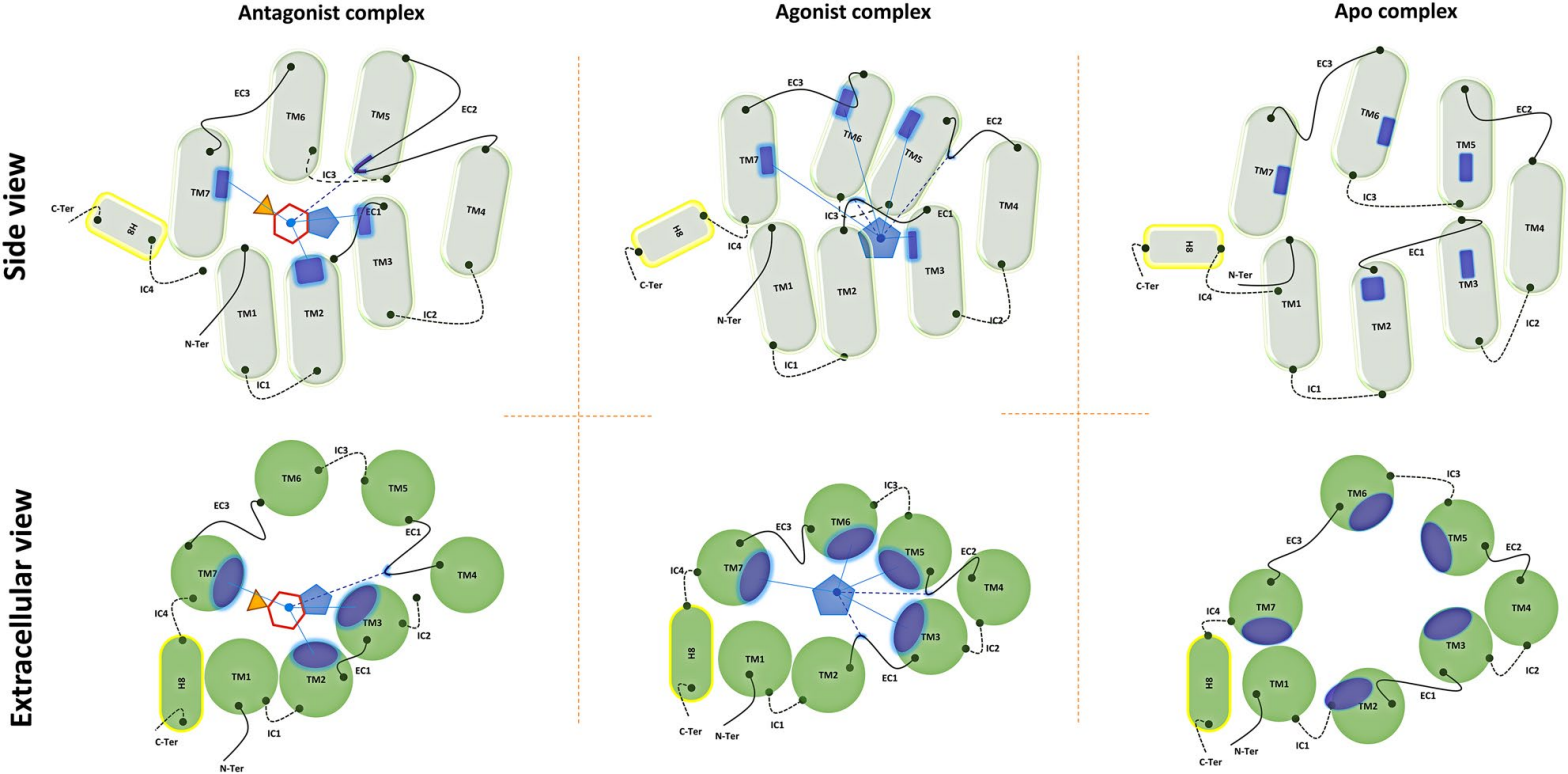
Structural differences between receptor states. Schematic representation of the structural differences between the inactivated, the activated and the constitutive-activity H3R. The blue patches represent the contacts between CPX (multi-polygon), HSM (pentagon) and the TM α-helices (cylinders or circles). They show the relative displacement and rotation of the helices between the different states. Continuous curves at the helix extremes show the extracellular loops and the dotted curves the intracellular loops. Straight (dotted) lines represent interactions between the ligand and the α-helices (loops). HME binding carries with it a reduction of the size of the site. Approximate tilts in the TM helices are shown in the side view. The relative orientation of the receptor among the three systems is the result of an optimal superposition of the TM helices.

Several new interactions are observed to contribute to the mechanism of signal propagation from the binding pocket to the G-protein binding sites in the cytoplasmic domain. Our results show that the biological state of the receptor is closely linked to its conformational dynamics and that the binding of a given ligand results in a variety of states, indicating the existence of ligand-induced heterogeneous receptor conformations, consistent with experimental data and the concept of protean agonism^24,57–61^. Thus, as observed in this work for H3R, functionally different ligands induce and stabilize distinct conformations in the receptor (Fig. 7), as opposed to the view based on the dynamic equilibrium of inactive- and active-state conformations.

We made other non-reported compelling observations. The high mobility in the binding site of the small endogenous agonist ligand HSM leads to several poses and eventually to HSM leaving the binding site after a certain time lag (Fig. 5). Before escaping into the solvent, it interacts with the N-ter and ECL2, consistent with the reports that ECL2 seems to be involved in ligand binding, selectivity and activation of GPCRs^62–64^, assuming the entry and exit pathways are similar. The pathway of ligand unbinding involves unfolding of the N-ter of the receptor. This very short-termed transition between the agonist-bound state and the constitutively activated state implies that the HSM-H3R complex is thus not a stable one and suggests that the orthosteric ligand can also act as an allosteric modulator. Experimentally, the agonist efficacy and the dissociation rate constant are highly correlated^65^, explaining the high efficacy of the HSM endogenous agonist. The time interval of the unbinding of HSM, ~ 2 ns, is very fast and fits together with the experimental rapid off-rates of agonists and their modest affinities^4,53^. Thus, in absence of interaction with the G protein cognate, the pre-active state obtained with the agonist-bound receptor is not stable^66^, leading in our situation to the permeation of HSM to the extracellular phase. In contrast, the binding of the antagonist was stable all along the trajectory. This dovetails with the experimental affinities measured for HSM and CPX, which indicate that the affinity of this latter for the receptor is at least an order of magnitude larger at the human H3R^12^. The unbinding pathway observed for HSM in this simulation suggests a plausible binding pathway in which HSM would bind to an extracellular vestibule of the receptor^39^, leading to transient activation, followed by “pre-activation” when accessing the orthosteric site, in a stepwise binding mode as part of a sequential activation^67^.

Otherwise, the H3R orthosteric site experiences significant plasticity because it can accommodate the conformations induced by functionally different ligands, in coherence with what is observed experimentally and computationally for many GPCR receptors^52,68^. Thus, the helices that are in contact with CPX are TM2, TM3 and TM7, as well as the ECL2 loop (Fig. 2, Table 1); for HSM, the corresponding helices are TM3 and TM7, as well as ECL2 (Fig. 4, Table 2).

Arrang et al.^12^ compared the potencies of H3‐receptor ligands on the inhibition of [^125^I]‐iodoproxyfan binding to rat and human H3R. CPX displayed significantly higher potency at the rH3R when compared to the hH3R. Production of a partially humanized chimeric rH3R allowed them to identify two residues responsible for this heterogeneity: Ala 3.37 (119) and Val 3.40 (122), both from TM3. In our structures of the CPX-rH3R complex, these two residues are not in direct contact with CPX (Fig. 2), suggesting short-range allosteric effects behind the experimental observations and an increased, favorable hydrophobic environment for rH3R, as compared to hH3R, which shows instead Val and Thr at positions 3.37 and 3.40, respectively.

The complexity of the atomic phenomena involved in the structure and dynamics of H3R, many of which cannot be observed experimentally, makes the design of ligands for the selective activation or inactivation of the receptor to produce the desired molecular and conformational effects challenging. Thus, the full amount of our results and the corresponding analyses lead us to believe that unraveling the mechanisms of signal transduction—activation, inactivation, constitutive activity—cannot be based on fluctuations in a single microscopic feature or a small number of them. These mechanisms are complex, as revealed in this work, and need multiple descriptors for better understanding. The interdependent, epistatic descriptors involve rigid-body motions of helices, along with variations in their mechanical properties:

- Multiple simultaneous rotamer toggle switches (Supplementary Figs. S16–S18).
- Ionic locks and inter-residue contacts (Supplementary Table S9).

We observed variations as well in other physicochemical characteristics, such as the:

- Formation and rearrangement of networks of charged residues (Supplementary Figs. S10–S12).
- Formation and rearrangement of H-bonds between amino acid residues (Supplementary Table S1), and between amino acid residues and internal water molecules (Supplementary Fig. S11).
- Differential composition of amino acid residues of the internal cavity of the receptor (Supplementary Table S2b).
- Presence of mobile networks of internal water molecules and their numbers (Supplementary Fig. S11).
- Presence of hydrophobic clusters (Supplementary Table S3).
- Specific lipid binding sites on the receptor (Supplementary Tables S4–S6, S8).

These complex properties may be the result of the size and multiple composition of the receptor and its environment (membrane, solvent, ions), and modulate its activity state^69^.

The dynamic properties of GPCRs may be modulated by the coupling to the membrane environment and its multiple structural states^70,71^ Finally, even though we observed distortions of the lipid bilayer for each system, their analysis was out of the scope of this work.

Besides confirming reported findings, the present work describes new phenomena that contribute to the mechanism of signal transduction, leading to signal propagation from the binding pocket to the G-protein binding sites in the cytoplasmic domain upon binding by the endogenous agonist, an inverse agonist, and in the absence of ligand binding. Altogether, we hope that our extensive study of the histamine H3 receptor through MD will contribute to a better understanding of signal transduction’s molecular events.

## Methods and materials

We used the Ballesteros-Weinstein generic numbering scheme for the amino acid residues of class A GPCRs^16,72^. The numbers attributed correspond to frame 0 of the trajectory.

### Homology modeling

We built the 3D structure of the transmembrane regions of the *R. norvegicus* histamine H3 receptor (Hrh3 gene, UniProt Q9QYN8, isoform 2 or rH3R(413)) with MODELLER’s sequence homology method^73^ by taking advantage of the diverse experimental X-ray structures of several homologous class A 7TM receptors that have become available in recent years, such as β2-AR (PDB codes 2RH1, 2R4R, 2R4S, etc.). Our use of a short isoform H3R is justified by the finding that the HSM auto-receptor is reported to be a short isoform of the H3R, that is, H3R(413)^74^. We paid attention by choosing active state structures as templates for the HSM-H3R and H3R apo homology models, and inactive state structure templates for the CPX-H3R homology model. Despite the lower-than-the-threshold (30%) overall sequence identity between H3R and other receptors needed to generate reliable 3D models, the high sequence homology per helix plus the pattern of highly conserved residues on each of the TM helices of GPCRs of class A allowed us to “anchor” the H3R sequence to the template sequences and thus use homology modeling with high confidence, as we are not sampling the folding of the protein. For example, the whole-sequence sequence identity between hH3R and hβ2-AR is ~ 22%, including ICL3 (~ 25% excluding ICL3), below the threshold generally used for homology modeling (~ 30%). However, we consider local, rather than overall, TM alpha-helix sequence homologies, as the large structural differences are localized in the extramembrane ends and loops. Thus, for TM1, for example, the local sequence identity between hH3R and hβ2-AR is 36%, as opposed to 22% for the whole sequence. The TM1-TM1 values of the rH3R:hβ2-AR pair are of 47.4% identity (89.5% similar, LALIGN software, EMBnet Server). We took the short isoform of the *R. norvegicus* sequence because its pharmacological characterization is more complete, and its sequence is 93.7% identical to *H sapiens*’.

As far as the loops connecting the TM helices is concerned, we employed a truncated form of the receptor by reducing the size of the IC3 loop (~ 140 residues), given its conformational heterogeneity and the fact that its long sequence shows no homology to any experimental 3D structure (a Blast search of ICL3 against the PDB gives no significant results). On another hand, a sequence analysis of ICL3 found several low-complexity regions, such as poly-Pro and poly-Ser, and sequence fingerprints or motifs. Nevertheless, its ab initio modeling is not possible due to its size. ICL3 has often been replaced by protein engineering to produce stabilized versions of the receptor for structure determination. Thus, when necessary, we replaced the fusion lysozyme from the template structures with several residues from the N- and C-termini of ICL3, resulting in a short loop. Some teams have tried to study the effects of ICL3 on β2-AR^75^. The starting conformation for ECL2, which contains about 22 residues, was unstructured; nonetheless, it folded into specific conformations as the simulations evolved. The other loops are of small size in the native sequence and we were in no need to generate their conformations. In all models obtained, the disulfide bridge between Cys 3.25 of TM3 and Cys 45.50 of ECL2 was satisfied. In addition, H8 in the C-ter was stable throughout the trajectory and remained in a cytoplasmic juxta-membrane-bound position, despite the lack of palmitoylation at Cys 8.59 and its insertion in the membrane. All these features indicate that our 3D homology models present a correct folding and are thermodynamically stable. For a more realistic and complete model, we added the N-ter of H3R to the agonist complex, not reported in experimental structures of homologous receptors, except for the crystal structure of the µ-type opioid receptor bound to an agonist in which a 13-residue fragment of the N-ter was left (residues 52–64)^76^. We achieved this by constructing first the corresponding peptide in an extended conformation and then fusing it to the N-ter of the crystal structure with PyMOL. Preliminary internal energy minimizations led the N-ter to fold back from the solvent in a stable conformation. The other two systems (antagonist complex and apo receptor) have a truncated amino terminus, like the µ-opioid receptor of Sounier et al.^66^ At the end, we selected for each system the model with the lowest value of the MODELLER objective function.

### Docking simulations

We generated three systems: two holo-receptors (an agonist–receptor complex and an antagonist-receptor complex), and an apo receptor, i.e. no ligand bound to the receptor. For the first complex, we used as the prototype ligand the endogenous agonist HSM in the (major) tele-tautomeric form (Supplementary Fig. S1a)—the quaternary amine at nitrogen N_ζ_ carries a positive charge. For the second complex, we took CPX as the prototype antagonist/inverse agonist (Supplementary Fig. S1b). The structures of the agonist-receptor and antagonist-receptor complexes were obtained after steered molecular docking calculations in the putative orthosteric binding site with the AutoDock Vina program^77^. For that purpose, we centered the grid box of about 30 Å of side length in the ligand pocket of the receptor, amid the TMs, ensuring thus that the search space was large enough for the ligand to translate and rotate; values for the exhaustiveness, num_modes and energy_range variables are of 12, 10 and 10, respectively. All single bonds in the ligands were free to rotate (two in HSM, and five in CPX). After docking of the ligand to the receptor, we further optimized by means of energy minimization the most representative complexes. We verified the stereochemical quality of the 3D models using the ProCheck^78^ and WhatIf^79^ programs. As far as the transmembrane regions is concerned, we estimate our 3D models to be of an accuracy corresponding to a ~ 3 Å resolution crystal structure.

### Molecular dynamics simulations

We used the Membrane Builder tool in the CHARMM-GUI server^80,81^ for construction of the membrane and for immersing the receptor in a pre-equilibrated symmetric lipid bilayer with the Insertion method. For the protein–membrane–water system, we generated a 1,2-Dipalmitoylphosphatidylcholine (DPPC)-based bilayer (PubChem CID 6138) in a rectangular water box in which we kept the ionic strength at 0.15 M by KCl. We used this salt as we wanted to see the effects of the monovalent potassium on the receptor, as opposed to sodium. The relative number of K^+^ and Cl^−^ counterions allowed us to obtain an electrically neutral system. The cubic box lipid bilayer consisted of about 188 phospholipids solvated with a shell of water molecules and ions placed around the bilayer with the program Solvate (https://www.mpibpc.mpg.de/grubmueller/solvate). We also included buried waters in the internal cavity of the receptor with the program Dowser (https://danger.med.unc.edu/hermans/dowser/dowser.htm). The space surrounding the bilayer was cropped to a rectangular, periodic simulation box with periodic boundary conditions. This configuration allows the receptor, placed in the center of the layer, and its periodic image, to be separated by a significant distance, thus avoiding unwanted receptor-receptor interactions. The dimensions of the box were of about 80 nm by 80 nm by 100 nm and depended on the system at hand. In addition to the receptor, the explicit water solvent, the phospholipids and the K^+^ and Cl^−^ counterions, the box contained, if appropriate, the ligand. The resulting systems contained 70,000–100,000 atoms. Among the several output files generated by the Charmm-gui.org server, we used the topology files for the subsequent MD simulations. The dielectric constant of the interior of the receptor is high and like that of the aqueous solvent (~ 78) due to the presence of internal waters.

We utilized the Propka^82^ program to assign the protonation states of the titratable groups of H3R at pH value 7.4. Nevertheless, we paid attention to the fact that the side chains exposed to the midst of the membrane are embedded in a low-dielectric, hydrophobic environment. Thus, the ionization states of ionizable side chains in the interior of the receptor (since it is saturated with water), in the phosphatidylcholine head-group zone and in the extra and intracellular zones were assigned as those corresponding to exposure to a high-dielectric surrounding. DPPC is a phospholipid that is well characterized physicochemically, whose experimental average surface area per headgroup in the gel phase is known^83^ (63 ± 1 A^2^ at 323 K). We performed MD experiments on a test membrane and noticed that it was neither necessary to apply any external surface tension in the calculations nor to impose the surface area per lipid, as we reproduced the experimental DPPC area per phospholipid in its absence. In addition, certain GPCRs have been shown to function in cholesterol-free membranes^40^.

We employed the Nanoscale Molecular Dynamics software package (NAMD), version 2.7b1, using the CHARMM-22/CMAP force field for proteins for the simulations. HSM and CPX were parametrized with the CHARMM General Force Field (CGenFF) program of the ParamChem initiative (https://www.paramchem.org) or with the CHARMM “patch” (https://www.charmm.org/charmm/documentation/by-version/c40b1/params/doc/struct/#Patch) command that allowed us to obtain the topology and parameter files.

We used the following parameters: Leapfrog Verlet algorithm for Newton’s equation integration; integration step of 2 fs allowing the SHAKE algorithm for keeping fixed all bonds involving hydrogen atoms; update of the lists of non-covalent pairs of atoms every 20 fs; long-range electrostatic interactions with the Particle Mesh Ewald algorithm, spacing 1 Å, 4 fs update; non-covalent cut-off radius of 10 Å and the non-bonded pair-list distance of 15 Å; microcanonical/NPT ensemble during equilibration and production runs; TIP3P model of water; temperature and pressure-coupled Langevin baths to ensure an isotherm and isobaric ensemble, with T = 323.15 K and P = 1.013 bar. The water thickness on either side of the membrane could be up to 35 Å.

We performed the simulation of the membrane-water system in three stages. During the first stage, we applied restraints on all non-solvent heavy atoms; we then energy-minimized the system for several hundred thousand steps using steepest-descent first and then conjugated gradient. Afterwards, we used several hundred thousand steps of conjugate gradient to minimize the side chains of the receptor and the aliphatic chains of DPPC, keeping the heads of the phospholipids and the backbone of the receptor fixed. Lastly, we used other several hundred thousand steps to minimize the free system with harmonic constraints on protein atoms, then by freeing all atoms. We followed this procedure by a slow warming-up of the system for 50 ps. In the second stage, we achieved full equilibration of the system by using the NPT ensemble for 100 ns, smoothly removing the applied constraints. The third stage of the simulation consisted of 800–900 ns production run under the NPT ensemble. We performed equilibration of the system in the absence of external pressure on the system, allowing energy dissipation without restriction.

We took the starting frame for all our analyses to be the frame at which the RMSD reaches a first plateau. For the antagonist complex, this corresponds to nanosecond 150, and for the agonist and apo receptors to nanosecond 112. Given the behavior of HSM, only the first segment of the agonist complex trajectory, when the ligand is well bound to the receptor, is considered for the analyzes (112–590 ns).

We ran most of the calculations in the massively parallel IBM Blue Gene of the HPC center IDRIS (https://www.idris.fr) in France.

## Supplementary information

Supplementary Information.

Supplementary Information.
