# Publisher Correction: Molecular dynamics of the histamine H3 membrane receptor reveals different mechanisms of GPCR signal transduction

**DOI:** 10.1038/s41598-021-81885-2

**Published:** 2021-01-25

**Authors:** Leonardo David Herrera-Zúñiga, Liliana Marisol Moreno‑Vargas, Luck Ballaud, José Correa‑Basurto, Diego Prada‑Gracia, David Pastré, Patrick A. Curmi, Jean Michel Arrang, Rachid C. Maroun

**Affiliations:** 1 UMR‑S U1204, Structure et Activité de Biomolécules Normales et Pathologiques, INSERM/Université d’Evry-Val d’Essonne/Université Paris-Saclay, 91000 Evry, France; 2 Computational Biology and Drug Design Research Unit, Federico Gómez Children’s Hospital of Mexico City, Mexico City, Mexico; 3 Laboratoire de Neurobiologie et Pharmacologie Moléculaire, INSERM U894, Centre de Psychiatrie et Neurosciences, 75014 Paris, France; 4 Área de Estudios de Posgrado e Investigación, Tecnológico de Estudios Superiores del Oriente del Estado de México, Los Reyes Acaquilpan, Mexico; 5 Laboratorio de Modelado Molecular y Bioinformática, Escuela Superior de Medicina, Instituto Politécnico Nacional, Mexico City, Mexico

Correction to: *Scientific Reports* https://doi.org/10.1038/s41598-020-73483-5, published online 09 October 2020

This Article contains a typographical error in the Results section. The subheading ‘Agonist and inverse aFree energy landscapes suggestgonist establish differential interactions with the receptor’

should read:

**‘**Agonist and inverse agonist establish differential interactions with the receptor’

