# Supplementary material for: Molecular dynamics of the histamine H3 membrane receptor reveals different mechanisms of GPCR signal transduction
Source: Sci Rep. 2020 Oct 9;10:16889. doi: 10.1038/s41598-020-73483-5 (PMC7547658; doi:10.1038/s41598-020-73483-5)
Supplement: Supplementary file 1 — Supplementary Information. [file 41598_2020_73483_MOESM1_ESM.pdf]

TITLE

# Molecular dynamics of the histamine H3 membrane receptor reveals different mechanisms of GPCR signal transduction

Short title: Molecular dynamics of H3R signal transduction


AUTHORS

Leonardo David Herrera-Zúñiga,[1,3,4] Liliana Marisol Moreno-Vargas,[2,3] Luck Ballaud,[3] José Correa-Basurto,[1,5] Diego Prada-Gracia,[2] David Pastré,[1] Patrick A. Curmi,[1] Jean Michel Arrang,[3] Rachid C. Maroun[1,3*]

[1] UMR-S U1204, Structure et Activité de Biomolécules Normales et Pathologiques, INSERM/Université d'Evry-Val d'Essonne/Université Paris-Saclay, 91000 Evry, France

[2] Computational Biology and Drug Design Research Unit, Federico Gómez Children's Hospital of Mexico City, Mexico City, Mexico

[3] Laboratoire de Neurobiologie et Pharmacologie Moléculaire, INSERM U894, Centre de Psychiatrie et Neurosciences 75014, Paris, France

[4] Área de Estudios de Posgrado e Investigación Tecnológico de Estudios Superiores del Oriente del Estado de México, Los Reyes Acaquilpan, Mexico

[5] Laboratorio de Modelado Molecular y Bioinformática, Escuela Superior de Medicina, Instituto Politécnico Nacional, Mexico City, Mexico

[*]Corresponding author: charbel.maroun@inserm.fr

General physical properties of the molecular system and their definitions

- The **root mean-square deviation** (RMSD) of the cartesian coordinates of the Cα atoms measures the structural drift of a molecule. In the absence of determining free energy landscapes for the membrane-protein system, the time dependence of the RMSD is a good indicator of the convergence of the 3D structure of the protein along the simulation to a stable state.

- The **RMSD matrices** contain the RMSDs using Cα atoms only between all possible pairs of structures from the trajectory. For generating the matrices, we used every 500th structure for the calculation. The RMSD color gradient goes from dark blue for small values, through yellow for medium values, to dark red for large values, with the origin at the upper left-hand corner. The different blue squares around the diagonal show time periods within which the structures resemble each other more than to frames outside these intervals. The blue squares may be taken to represent subfamilies or ensembles of conformations. The passage from one square to the other indicates different conformer families. The local conformational transitions involved in these changes in RMSD are reflected in the high number of blue regions around the diagonal of the corresponding RMSD matrix.

- The **2D distance or contact maps** for the average structure represent the Cα atom distance between all possible amino acid residue pairs in a 3D protein structure. They can be used to describe similarity between protein structures and to represent characteristic patterns of secondary structure. The origin, i.e., residue 1 (N-ter) for the x- and y-axes is at the upper-left corner. The last residue (C-ter) is at the lower-right corner. Red zones denote proximities between atoms, and thus between helices.

- **Principal Component Analysis** (PCA) represents a classic dimension reduction approach by constructing orthogonal linear combinations of many properties (in this case conformations), called principal components (PC). The greatest variance of the data lies on the first component, the second greatest variance on the second component, and so on. The PCA allows the identification of conformational communities or clusters, dividing the conformations into several populations and covering in this way a well-defined region in diversity space given by the principal

components. These essential degrees of freedom describe major collective modes of fluctuation that are relevant for the function of the protein.[1] PCs may be referred here to as 'meta- or super-conformations' and are very useful in investigating the molecular motions of proteins.[2,3] Through the PCA, molecular dynamics simulations (MD) allow the sampling and the grouping of similar conformational states.

For the analysis of the data generated by the MD production trajectories, we used the following programs:

- EUCB[4] for the computation of the following properties, with 10 ns jumps and a switch distance of 5 Å.
    - Root mean-square fluctuations (RMSF) of each amino acid residue
    - H-bonds
    - Ionic pair matrices or charged-residue clusters
    - Conformational switches
    - Side chain rotamer angles
    - Ionic bridges
- SIMULAID[5,6]
    - H-bonds between protein and solvent
- CARMA[7]
    - Root mean-square deviation (RMSD) of the cartesian coordinates of Cα atoms with respect to the initial frame
    - RMSD matrices between structures
    - Distance maps
    - The principal component analysis (PCA) of the Cα atoms of the protein

- PCA-based free energy landscapes or ΔG plots obtained from the equation $\Delta pG = -k_B T \ln(P/P_{max})$, where P and Pmax are probabilities obtained from the distribution of the principal components for each structure (frame) of the trajectory.

- VMD[8] and Tcl/TK home-brewed scripts run in VMD
  - Distances between amino acid residues
  - Detection of the residues of the internal cavity of the receptor
  - Non-covalent contacts between cavity residues and ligand
  - Distribution and residence times of lipids around the protein
  - Lipid binding sites on the protein
  - Positions and residence times of water molecules in the interior of the protein
  - H-bonds between residues
  - Visualization of the trajectories
  - Production of molecular figures
- PyMOL (PyMOL Molecular Graphics System, Version 2.0 Schrödinger, LLC.)
  - Molecular graphics and visualization

The residues composing the internal cavity of the representative structures of the receptor produced by CARMA were defined by finding the "wet" residues, i.e. those residues that were in contact (3 Å or less) with an internal water for more than 80% of the trajectory. The "wet" atoms of the ligands were determined in the same fashion. This approach determined the residues that had an interaction with a water molecule that had itself an interaction with the ligand. On another hand, pocket detection, ligand binding site and analysis and visualization of tunnels and channels were performed with the Fpocket,[9] SiteHound[10] and Caver software tools.[11] Those residues found in at least three out of four searches were considered cavity residues.

We did not deem necessary to energy-minimize the complexes at the frames we looked at during the analysis. Instead, we selected ten frames at regular intervals of the MD trajectory to obtain the interactions between the ligand and the receptor, since we compiled the long-lasting neighbors of the former all along the trajectory.

When dealing with the lipid components of the membrane, we wished to find out whether there were any DPPC-specific binding sites on the receptor. For this purpose, we found out the amino acids that are in contact at least 80% of the time with at least one atom of a lipid molecule defining high-affinity, specific binding sites with the membrane protein.

We performed the computations in a Linux cluster with one master node of 8 CPUs, 10 To stocking; and 192 CPUs (16 nodes of 12 Intel XEON E5630@2.53GHz, 24 GB RAM, CPUs each). The NAMD parallel jobs were executed through the mpiexec application. We also ran calculations in the massively parallel IBM Blue Gene of the HPC center IDRIS (http://www.idris.fr) in France.

## RESULTS

### - RMSD plateaus of the MD trajectories imply physically stable systems

Fig. S1a presents the chemical structure of the endogenous agonist histamine (HSM), and Fig. S1b that of the inverse agonist ciproxifan (CPX). Fig. S2 shows the RMSDs of the Cα atoms with respect to the initial structure. For the **antagonist-H3R** complex, the RMSD attains a plateau at about 150 ns, remains constant at 4.2 Å until ~550 ns and then rises slightly to 4.8 Å. The **agonist-H3R** complex shows an RMSD that attains values of ~5.0 Å at 600 ns until the end of section 1. This observation is consistent with the fact that agonist ligands tend to be less efficient in the stabilization of the structure of the protein.[12] The **apo** receptor shows considerable changes in the RMSD, especially in the middle of the trajectory, just before 600 ns, when it reaches almost 5 Å of RMSD, to fall afterwards to around 3.5 Å at the end of the 930 ns trajectory. All in all, the observed fluctuations suggest a good packing quality of each model.

For the structurally-conserved TM helices of the receptor, the RMSDs of each of the three systems with respect to the crystal structure of the H1R-doxepin complex at 3.1 Å resolution[13]

(PDB code 3RZE) are as follows: 3.1 Å for the last frame of the antagonist-H3R complex, 2.5 Å for the last frame of the agonist-H3R complex, and 2.0 Å for the apo receptor. These data indicate that the MD trajectories lead to physically stable systems.

- RMSD matrices point to high structural variations

Fig. S3a for the **antagonist-H3R** complex shows a tendency for the blue areas to get darker as the trajectory unfolds, indicating that subsets of similar conformations persist. For the first period (section 1) of the **agonist-H3R** complex (Fig. S3b), i.e. when histamine is bound to the receptor (up until nanosecond 590), the structures up to 328 ns resemble each other a lot (small RMSD values) at the beginning of the first phase of the period. Then about five subsets of conformations appear, of which the last one is maintained sometime after histamine unbinding. Then another subset appears centered around 726 ns. For the **apo** receptor (Fig. S3c), the RMSD matrix is rather sparse and weak in blue regions, i.e. in small values of RMSD, indicating higher structural variations.

- Cα-Cα distance maps indicate that the overall relative positions of TM helices in the apo receptor and the agonist-H3R complex are equivalent

The Cα-Cα distance map of the average structure of the **antagonist-H3R** complex shows that CPX (represented in the map after all the amino acid residues) is in contact with TM2, the N-ter of TM3, ECL2, the C-ter of TM6 and the N-ter of TM7 (Fig. S4a). For the first section of the trajectory of the **agonist-H3R** complex, the proximity relationships are in Fig. S4b and for the **apo** receptor in Fig. S4c. All relative positions of the helices are shown in the form of a matrix for each of the three systems in Fig. S4d, in which an x represents the mentioned proximities between TM helices. The matrices are symmetrical. We can see that the relative TM helix positions of the average structure of the agonist-H3R complex and apo receptor are similar, with a clear off-diagonal line showing a sequential array of proximities: TM1-TM2-TM3-TM4-TM5-TM6-TM7. The inter-helix distribution of spatial distances for the antagonist-H3R complex shows no proximity between TM2 and TM4, TM3 and TM7, and TM4 and TM5; instead, this complex shows a proximity between TM2 and TM6. As mentioned in the Introduction, H3R possesses a high constitutive activity in the absence of agonist. Our results

show indeed that the overall relative positions of TM helices in the apo receptor and the agonist-H3R active complex resemble each other. In addition, ECL2 is in contact with the antagonist, in conformity with the importance of this loop in ligand binding, selectivity and (in)activation,[14] as well as stabilization of the inactive state of the receptor.[15]

- Eigenvectors show intra-molecular correlated movements and inter-molecular differential movements.

The eigenvectors represent the global displacement of each residue in the system for all the trajectory and are used to generate new conformations. The images in Fig. S5 show the superposition of these conformations for each of the four clusters (C1-C4) generated by the PCA for the **antagonist-H3R** (Fig. 1a) complex. The ensemble of conformations in Fig. S5a corresponds to cluster 1 (C1) and shows that this mode involves essentially movements of the N-ter extracellular segment of the receptor and its ECL2, which act as a flexible lid that opens and closes on top of the ligand-binding cavity. Loops ICL2 and ECL2 exert a push-n-pull motion on TM4 as a rigid body. For TM5, we observe a compression-extension movement of the fragment above Pro 5.50 and a kink movement around this residue. We also observe kink movements around Pro 6.50 and Pro 7.50 of the conserved $^{6.47}$CWXP$^{6.50}$ and $^{7.49}$NPXXY$^{7.53}$ motifs, respectively. All these movements are correlated. No other important movements are detected in the other helices. Cluster C2 (Fig. 1a) shows large correlated movements of ECL2, ICL2 and to a lesser extent ICL3, with slight kink motions of TM1 and TM5-TM7 around their middle-helix proline residues (Fig. S5b). In addition, TM5-TM7 move collectively in a breathing motion along the plane of the membrane. The mode corresponding to cluster C3 (Fig. 1a) shows large amplitude motions for ECL2, and a pendulum-like rigid-body motion of the lower zone of TM5-7 with their IC loops (Fig. S5c). Fig. S5d shows the collective modes of movement corresponding to cluster C4. First is an angular N-ter to C-ter motion of H8, whose C-ter gets close and far from the membrane, accompanied by a concerted motion of the N-ter, ECL3 and ICL2, indicating long-range communication between the extra- and intra-cellular regions of the receptor. With regards to the eigenvectors in the **agonist-H3R** complex (fig. 1b), Fig. S6a shows the conformations of cluster C1 with correlated motions of the N-ter and ECL2 extra-cellular segments of the receptor, in agreement with experimental 3D structures of active state GPCRs, such as β2-AR.[12,16–18] Along these motions are conformational fluctuations in

ICL2, leading to an opening and closing of the far-away intra-cellular domain of the receptor. Fig. S6bcd show that motions are dominated by the N-ter of the receptor. Indeed, in activation of the μ-opioid receptor, the binding of agonists leads to conformational changes in the amino terminus that makes contact with the 7TM bundle to cap the binding pocket.[19] Fig. S7a shows the eigenvectors of C1 for the **apo** receptor. The N-ter, ECL2 and ECL3 undergo large concerted unfolding-folding motions, with ECL2 exerting a hinge-bending motion on TM4 perpendicular to the plane of the membrane. For C2 (Fig. S7b), the motions are much reduced. For C3 (Fig. S7c), large motions of N-ter and ECL2 are observed again, accompanied of an important movement of the ICL3 loop.

In conclusion, the differences in morphology of the clusters of the holo-receptor (Fig. S5, Fig. S6) with respect to those of the apo receptor (Fig. S7) suggest that receptor conformations are ligand induced, and that the N-ter and ECL2 fragments of the receptor fold on top of the binding cavity of the holo-receptor.

- Clusters of charged residues get reconfigured from one state of the receptor to the other

Electrostatic interactions are of special importance for membrane proteins because of the low dielectric environment in membranes. We thus proceeded to detect clusters of charged residues using a distance criterion of 12 Å between COMs of the side chains of amino acid residues.

For the **antagonist-H3R** complex, there are two charged amino acid clusters that persist 90% of the time of the production trajectory. One cluster is in the extracellular region of the receptor and the other one in the intracellular region. The two clusters are represented in Fig. S8. The amino acid residues for the first cluster belong to TM3, ECL2, TM5-TM7, and those of the second cluster to TM1, ICL1, TM3- TM6, ECL1, ICL2, ICL3 and H8. For the **agonist-H3R** complex, section 1, two independent clusters persist while HSM is bound to the receptor (Fig. S9). They involve N-ter, TM3, ECL2, TM6 and TM7 for the first network; and TM3- TM6 for the second. One residue of the first cluster, Asp 3.32, is in contact with the protonated amine (pKa ~9.4) of the aliphatic amino group of HSM, consistent with experimental data.[20] Four clusters of charged residues can be found for the **apo** receptor (Fig. S10). Amino acid residues from TM1, TM3 and ECL2 compose the first cluster; one residue from each TM3, 5 and 6 the second cluster; residues from the N-ter, ECL1 and TM6 the third cluster; and residues from TM1, ICL1,

ICL2, TM3-TM5, ICL3, TM7 and H8 the fourth cluster. Many of these residues are also involved in long-lasting H-bonds, i.e. prevailing more than 70% of the time.

- H-bonded networks between amino acid residues are a function of the state of the receptor

For the **antagonist-H3R** complex, inter-residue H-bonds with residence times greater than 70% connect ECL2 (TM4-TM5 loop) and ICL2 (TM3-TM4 loop) to TM3; N-ter to TM7; TM1 to H8; and ECL2 to ECL2 (Supplementary Table S1). For the first segment of the production trajectory of the **agonist-H3R** complex, ECL2 is H-bonded to the N-ter, TM3 and TM4; and the N-ter of TM1 to H8 (Supplementary Table S1). Finally, for the **apo** receptor, only one non-intra-helical H-bond persists (Supplementary Table S1). Thus, it is interesting to notice that the antagonist-bound receptor shows the largest number of inter-residue H-bonds, providing the structure of this complex with an added energetic and structural stability.

- The composition of the internal cavity of the receptor abounds in Tyr and Leu

Supplementary Table S2b shows that there are 38 residues that form the orthosteric cavity of the **CPX**-bound receptor. These residues are contributed by TM2, TM3, ECL2, and TM5-TM7. The environment of the ligand is rather hydrophobic with the three aromatic amino acid side chains being represented. Twenty-six residues compose the internal cavity of the **agonist**-H3R complex, with contributions coming this time from TM1, TM3, ECL2, and TM5-TM7. For the **apo** receptor, the cavity is composed of only 24 residues. As expected, this cavity is the smallest one, given the absence of ligand and the subsequent contraction of the orthosteric cavity. Secondary structures contributing to it are TM2, TM3, ECL2, and TM5-TM7. In all three systems, TM4 being eccentric, it contributes with no residues to the receptor's cavity, just like all loops (except ECL2) and, of course, H8 (the short C-ter juxta-membrane helix in the cytoplasmic side). Notice the contribution of ECL2 to the morphology of the binding cavity.

The residues that compose the internal cavity of H3R in its different states are of diverse types, with Tyr and Leu being the most abundant, followed by Phe and Ser. Residues Pro, Gly, Gln, Lys, Thr and His are absent (Supplementary Table S2b). Interestingly, the antagonist and agonist pockets show a net negative charge of 4, whereas the net charge of the pocket of the

apo receptor is neutral (Asp 3.32 + Arg 6.58). The cationic ligands HSM and CPX thus induce changes in the pocket that bring into play acidic residues.

- Water molecules in the internal cavity and their H-bonded networks are persistent

A persistent dynamic network of water molecules is observed in the interior of the receptor, with waters penetrating and exiting the receptor from the extracellular region. These waters establish several H-bonded networks; nevertheless, the receptor is not a water channel -it presents a molecular plug formed by the lower-leaflet internal walls of TM2, TM3, TM6 and TM7, and the TM7-H8 loop that keeps the internal water molecules from exiting to the cytoplasm or cytoplasmic bulk waters from entering the receptor. About 61 water molecules are constantly present in the internal cavity of the **antagonist**-bound receptor during the trajectory, 35 for the **agonist** one, and 49 for the **apo** receptor (Supplementary Table S2a), so that water penetration to the receptor is largest upon antagonist binding, and smallest for the agonist-H3R complex, just like in the high-resolution structure of α2A-AR that reveals about 60 internal waters.[21] In addition, Yuan et al.[22] mention an increased penetration of water into the receptor cavity of μ- and κ-opioid receptors, which has been linked to the activation mechanism upon agonist binding. Therefore, the presence of water in the binding site clearly demonstrates its influence in the dynamics and conformation of the receptor.

We determined the H-bonded networks established by water molecules in the cavity with occupancy greater than 75%. In all three systems, these occupancies are characterized by very low residence times, of the order of 20% maximum, indicating a high fluidity of water molecules. Thus, even if a site may be continually hydrated, it is not so by the same water molecule 4/5 of the time. Again, the observation of the itinerary of the water molecules indicates that these enter and exit the internal cavity of the receptor through the extra-cytoplasmic region of the receptor only, no water molecules neither entering nor exiting the receptor through the cytoplasmic region. The internal cavity of the receptor has the shape of a bent funnel with a flexible lid at the top composed of the N-ter and the ECLs, especially ECL2.

For the **antagonist-H3R** complex, residues that contribute with their side chains to H-bonded networks in the cavity zone are in Fig. S11. Of these, Asp 2.50, Glu 5.46 and Ser 7.46 form two or more H-bonded bridges. The two largest networks involve five waters, Asp 2.50 and Ser

7.46 the first (Network 5); and four waters, Glu 5.46 and Thr 6.52 the second (Network 6, Fig. S11). Finally, the interaction of CPX with two residues in the protein, Tyr 2.61 and Asp 3.32, is mediated by two water molecules for the former, and by one for the latter (Network3, Fig. S11). Notice that TM1 and, of course TM4 and H8, do not participate to the H-bond network during CPX binding. The only charged amino acids in the interior of the receptor are in the binding cavity and are Asp 2.50, Asp 3.32 and Glu 5.46 (Fig. S11). The **agonist-H3R** complex shows two networks of H-bonds, the first of which includes HSM bonded to a water molecule and to Asp 3.32 (Fig. S11). The two networks of the **apo** receptor involve, each one, two internal water molecules (Fig. S11).

- Hydrophobic clusters may contain up to seven residues

We report in Supplementary Table S3 the hydrophobic clusters at 90% occupancy formed by at least three side chains for the antagonist-H3R complex, the agonist-H3R complex and the apo receptor, respectively. The cutoff used is of 6 Å between the COMs of each amino acid residue; the set of residues is composed of Ile, Leu, Val, Phe, Met, Cys, Trp, Pro and Ala.

For the **antagonist-H3R** complex, there are six clusters. The distribution of clusters is as follows: two clusters of three residues, two clusters of four residues, one cluster of six residues and one cluster of seven residues. One cluster, implying the N-ter and ECL1 contains 7 residues (Ala 23 from the N-ter, Phe 2.56, Cys 2.57, Leu 2.60, Trp 23.50 (ECL1), Leu 3.24 and Trp 3.28). For the **agonist-H3R** complex, there are six clusters, and for the **apo** receptor there are five clusters. These non-covalent interactions, just like the H-bond networks, depend on the state of the receptor.

- Side-chain switches for the Met residues are not detected

Kofuku et al.[23], by monitoring the NMR signals, investigated the role of Met residue 82 (2.53) in antagonist- and partial agonist-bound states of the β2-AR, which are correlated with conformational changes of the transmembrane regions upon activation. The corresponding residue in H3R is a valine and none of its neighbors is a methionine; nevertheless, we decided to monitor the conformational states of all Met residues for the three systems. We found that

in all three systems, all but one of the methionine residues remain in the t or g- states throughout the trajectory. As opposed to the β2-AR then, the conformational states of the methionine residues remain unchanged between the apo receptor, and its antagonist- and agonist-bound states; there is thus no correlation of conformational changes of the methionines upon activation in H3R.

- Detection of lipid binding sites on the receptor leads to amino acid sequence motifs

We list in Supplementary Table S4 the DPPC lipids attached to the receptor in the **antagonist** state and the amino acid residues they interact with. The residence time of a given lipid is listed in the first line of the "occupancy" column; the next lines show the residence times of the lipid with different amino acid residues of the receptor.

In résumé, for the **antagonist-H3R** complex, the structures in contact with the 13 lipids are TM1, ECL2, TM3-TM6, ECL3, TM7, TM7-H8 loop, and H8 (Fig. S12, Supplementary Table S4). For the **agonist-H3R** complex, the following secondary structures participate to the binding of the seven lipids: ICL1, TM2, TM5, TM6 and the TM7-H8 loop (Fig. S12, Supplementary Table S5). Interestingly, for the **apo** receptor the 5-lipid binding site involves all helices except TM5 (Fig. S12 and Supplementary Table S6).

Supplementary Table S7 shows the amino acid residues of the receptor in contact with lipids for each of the three systems grouped. First, the fraction of non-polar residues (43-56%) is the largest, followed by the aromatic residues (15-26%), and the polar and charged classes. The most frequent residue of all groups and for all three states of the receptor is leucine. In the second class, Phe is the most abundant for the antagonist and agonist (section 1) systems, and Tyr and Trp for the apo system. In the polar group, Ser and Thr for the antagonist**-** and agonist-H3R complexes are the most represented residues. Finally, the antagonist-H3R complex is the system showing the most contacts of charged residues with lipids -eleven Arg and six Lys. The non-polar and aromatic class residues are in contact with the larger area acyl-tails of the lipids; whereas the basic residues interact with the negatively charged lipid head groups.

Supplementary Table S4 for the **antagonist** complex shows 13 highest-occupancy lipid molecules associated to the receptor, of which four are in the upper-leaflet of the membrane

and nine in the lower-leaflet (Fig. S12). As an illustration of the interaction of DPPC lipids with the inactivated H3R, Fig. S13 shows a 2D LigPlot+ diagram of lipids 125 and 149, showing H-bonds between Arg 8.51 and one oxygen atom from the phosphate head, and hydrophobic interactions with surrounding residues for lipid 149. Both lipids share Leu 7.55 as an interacting residue. In Fig. S14, extra- and intra-cytoplasmic views of the inactivated receptor for the 490 ns frame, we can see that DPPC molecules essentially bind to opposite sides of the exposed surfaces of the receptor. On one side of the receptor, TM1, TM6 and TM7 lodge three lower-leaflet lipids and one upper-leaflet lipid. The other side, with TM3, TM4 and TM5 offers another binding site for two lower- and two upper-leaflet lipids. On that side, an upper-leaflet lipid finds a binding site provided by TM3 and TM4. Both leaflets contribute thus with lipids for receptor binding. For the **histamine** complex, all in all, there are five lipids in the lower part of the membrane, one in the upper part, and one in the middle (Fig. S12, Supplementary Table S5). A LigPlot+ diagram illustrates the surroundings of lipid 2 (Fig. S15a). The Lys residues interact with the phosphate moiety of the DPPC molecule, whereas the hydrophobic residues with its fatty acyl tail chains. For the **apo** receptor, there are five lipids binding to sites in the upper leaflet of the double bilayer (Fig. S12, Supplementary Table S6). The LigPlot+ representation of Fig. S15b shows lipid 184 and its neighboring residues, all but one belonging to TM7. Nε1 of Trp 7.40 interacts with O22 of the DPPC lipid; Leu 1.42 and Ala 7.47 interact with one of the acyl tails of the lipid. Many water molecules surround the phosphate and choline moieties.

In order to extract the lipid binding H3R amino acid sequence motifs, we selected those amino acid residues in Supplementary Table S4 to Supplementary Table S6 in contact with lipids that show residence times ≥ 90%. The resulting binding motifs are in Supplementary Table S8.

- Leu is the most frequently membrane-exposed residue

The fraction of membrane-exposed hydrophobic amino acid residues is of 71, 71 and 67% for the antagonist, agonist (section 1) and apo systems, respectively, with Leu being the most frequently exposed residue, representing 27-29% of all membrane-exposed residues. With respect to all exposed residues, the hydrophilic residues, represent 22%, 12%, and 18% for the antagonist complex, respectively. As far as the charged residues is concerned, their relative

exposed populations are of 8%, 17% and 15%, for the antagonist complex, for the agonist complex and for the apo receptor, respectively.

- Rotamer toggle switches are multiple and concerted

The goal of this section is to determine those amino acid side chains that undergo concomitant side-chain conformational changes. We focus on the aromatic side chains of Tyr, Trp and Phe, since several rotamer toggle switches dealing with those residues have been reported in the literature.[24]

Fig. S16 shows, for the **antagonist-H3R** complex, the time evolution of aromatic side chain dihedral angles $\chi_1$ (N-Cα-Cβ-Cγ) that undergo side chain conformational transitions. We can see that $\chi_1$ of Trp 6.48 of the CWXP ($^{6.47}$CysTrpXPro$^{6.50}$) motif adopts values of -60° (g- configuration) most of the time, until 665 ns, when it undergoes a transition to a trans conformation (±180°). Concomitantly, $\chi_1$ of Trp 1.31, Trp 45.36 and Phe 5.47 undergo correlated conformational transitions. Moreover, another concurrent $\chi_1$ change for Phe 3.51 and Trp 7.43 can be observed earlier, at around 615 ns (ribbon model in Fig. S16). Most of these residues are in the upper half of the receptor (towards the extra-cytoplasmic zone), suggesting that, at this stage of the mechanism, conformational changes in this zone may be enough to transmit the signal to the lower half of the receptor. Given that several of these side chains are rather far from each other, it seems that other side chains mediate the transmission across the membrane. As compared to the MD simulations of Nygaard et al.[25] (their Fig. 5a), Trp 6.48 (TrpVI:13) in our extended MD simulations adopts an intermediate conformation between the active and inactive states. For instance, starting at about half of the trajectory, the average distance between the center of masses (COMs) of the Phe 5.47 (Phe V:13) and Trp 6.48 side chains is ~13 Å. Tyr 7.53 of the NPXXY motif ($^{7.49}$AsnProXXTyr$^{7.53}$) is in the active conformer state throughout the trajectory (not shown), showing no switch; it engages in a hydrophobic interaction with Ile 6.40[25] (their Fig. 5b). Thus, in a remarkable fashion a multiple toggle rotamer switch of certain aromatic chains, along with bi-modal switches appears to be important for receptor H3R inactivation. For the **agonist-H3R** complex (section 1), the conformation of $\chi_1$ of Phe 5.47 remains constant at g- and Phe 3.41 undergoes a g- to trans transition in the last fourth of the trajectory (not shown). Concomitant transitions

in $\chi_1$ of several aromatic side chains take place starting at about 0.5 µs (Fig. S17). Again, a multiple toggle rotamer switch of aromatic side chains takes place during H3R activation and involves several amino acid residues. Only Trp 6.48 is common to both mechanisms - activation, inactivation. All but one of the residues are in the upper zone of the receptor and imply the ECL1 and ECL2. The number of aromatic side chains that undergo conformational transitions in the **apo** receptor is much reduced and includes only Phe 45.54 and Tyr 45.56 of ECL2, and Tyr 7.33 and Phe 8.54 side chains, with the first and the third toggle switches interconverting between g+ (+60°), g- (-60°) and trans states, and the second and third toggle switches common to the activation mechanism (Fig. S18). The mentioned residues are in the upper and middle zones of the receptor, except for Phe 8.54, belonging to H8. None of the residues of the DRF ($^{3.49}$AspArgPhe$^{3.51}$) motif are thus involved in H3R activation or constitutive activity implicating that the ionic lock involving Arg3.50-(Asp 6.30 and Asn 5.64) is not conserved. This is consistent with the lack of direct evidence for a role of that motif in those two situations.[26]

In résumé, there are multiple concerted long-range rotamer switches for each system as the involved residues are several tens of angstroms away from each other. Finally, it is interesting to observe that aromatic side chains switches from ECL2 participate in the different mechanisms.

- Inter-residue contacts are unique to each state of the receptor; novel ionic locks are detected

We have measured many selected inter-residue distances using the COMs of the side chains to detect contacts. Those pairs of residues interacting according to the criteria described in the M&M section are in Supplementary Table S9 for each of the three systems. Several of those interactions represent ionic locks.

For the **antagonist-H3R** complex, the conserved arginine of the ionic lock (motif DRF) among rhodopsin-like G protein-coupled receptors, Arg 3.50, interacts with Asp 3.49 and Asp 6.30 in a constant but dynamic fashion throughout the trajectory, in agreement with the behavior of the ionic lock in the MD simulations of the β2-AR receptor.[27] In addition, there is formation of a hydrophobic interaction between Met 1.39 and Trp 7.40/Trp 7.43, followed by disappearance of an initial Met 1.39-Tyr 2.61 interaction; the interaction between Met 6.55

and Tyr 3.33/Phe 7.39 is sporadic. Lastly, the distribution of the Met 6.55-Tyr 6.51 distance with a major peak at 5 Å and a minor one at 9 Å is analogous to one described in the literature (Nygaard et al 2013, their Fig. 6C).[28] For the **agonist-H3R** complex, the following interactions emerge: Met 1.39-Trp 7.40, Met 6.55-Phe 5.48, while the Phe 5.47-Trp 6.48 distance becomes distended; Met 6.55-Tyr 6.51/Tyr 3.33, and Tyr 7.53-Phe 8.50 distances are constant throughout. For the **apo** receptor, are stable throughout the trajectory the following contacts: Asn 1.50-Pro 7.50, Asn 2.39-Asp 3.49/Arg 3.50, Asp 3.32-Trp 7.43, Asp 2.50-Pro 7.50, Met 6.55-Tyr 6.51, Met 1.39-Tyr 2.61/Trp 7.40/Trp 7.43, Met 1.54-Phe 2.51, and Trp 6.48-Ser 7.46. A Met 6.55-Tyr 3.33 interaction goes away gradually. Those distances that undergo a notable change include formation of interactions Phe 5.47-Trp 6.48, and Tyr 7.53-Phe 8.50/Phe 8.54; and intermittent interactions Arg 3.50-Asn 2.40/Asp 3.49/Asp 6.30, and the Met 4.46-Trp 4.50 couple.

In conclusion, only the apo receptor presents interactions between TM1 and TM2, and between TM2/TM3 and TM7. Interactions between TM3 and TM6 are unique to the agonist and antagonist-H3R complexes. Asp 3.32 in the apo receptor not being involved in interaction with a ligand interacts now with Trp 7.43. Notice that the Arg 3.50-Asp 3.49/Asp 6.30 H-bond and electrostatic interaction is unique to the antagonist-H3R complex. The Asn 2.39-Asp 3.49/Arg 3.50 interaction is absent in the agonist-H3R complex, just like interactions between TM7 and H8 are absent in the antagonist-H3R complex (not shown).

- Diffusion of a potassium monocation into the antagonist complex sodium allosteric site.

$K^+$ begins its course towards the interior of the receptor between TM5 and TM6 and is in contact with Leu 5.39 and His 67.00 (ECL3); it is hydrated at this time by five water molecules. A short interval after, it interacts with Ser 5.43, Glu 5.46, Phe 5.57 and Met 6.55 from TM5 and TM6, and is coordinated by only two to three water molecules. Towards the end of its path, it establishes contacts with Asp 2.50, Cys 3.36, Ser 3.39, Glu 5.46 and Trp 6.48 (TM2, TM3, TM5 and TM6), several of which are mediated by water, like with Asp and Glu residues, to finally become rehydrated by five water molecules. From time to time, the monocation is in contact with the highly conserved Asp 3.32 of the binding pocket. In the last frame of the trajectory, the cation is coordinated by Asp 2.50, Asp 3.32 and by three structured water

molecules (r<3.0 Å), just like in the $\alpha$2A-AR.[21] As opposed to HSM, its insertion in the pocket is irreversible in the time scale studied (Fig. S19, Fig. S20).

FIGURES

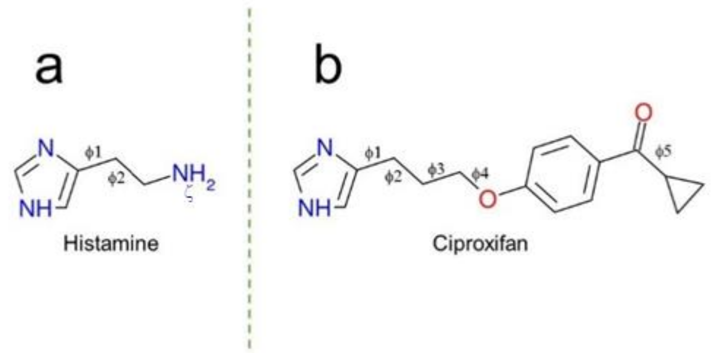


Fig. S1. Chemical Structure of a) histamine (HSM), and b) ciproxifan (CPX), with torsions around single bonds denoted ϕ.

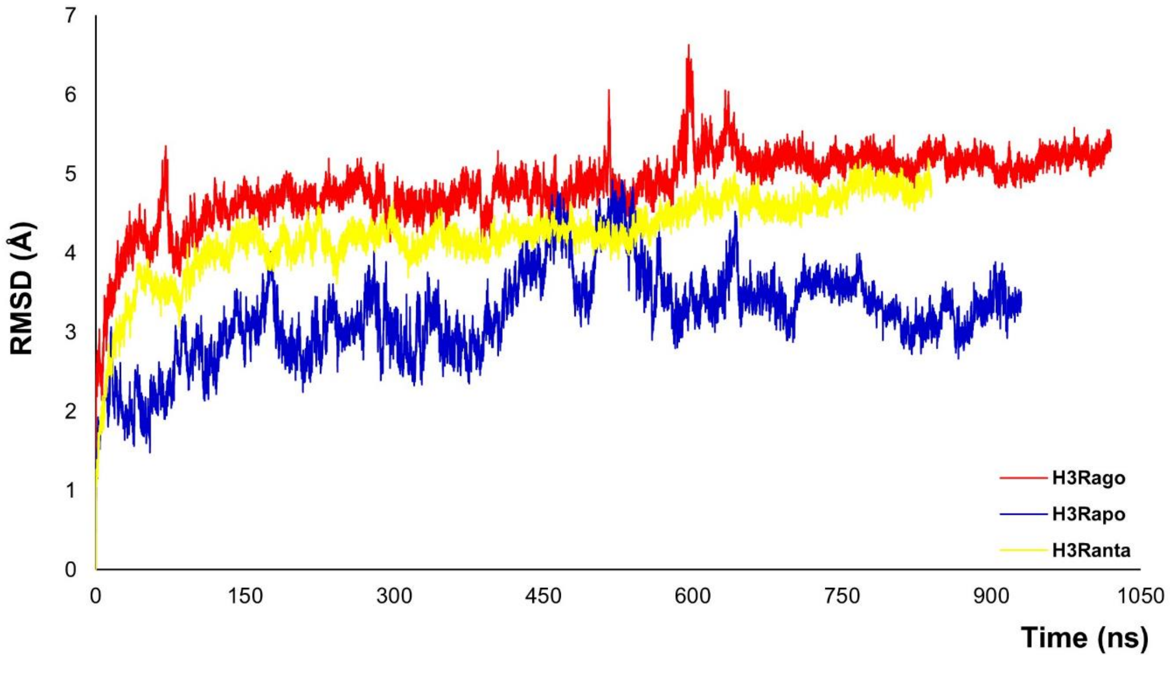


Fig. S2. Time-dependent Root Mean Square Deviation (RMSD) with respect to the initial structures for the antagonist (yellow), agonist (red) and apo (blue) structures.

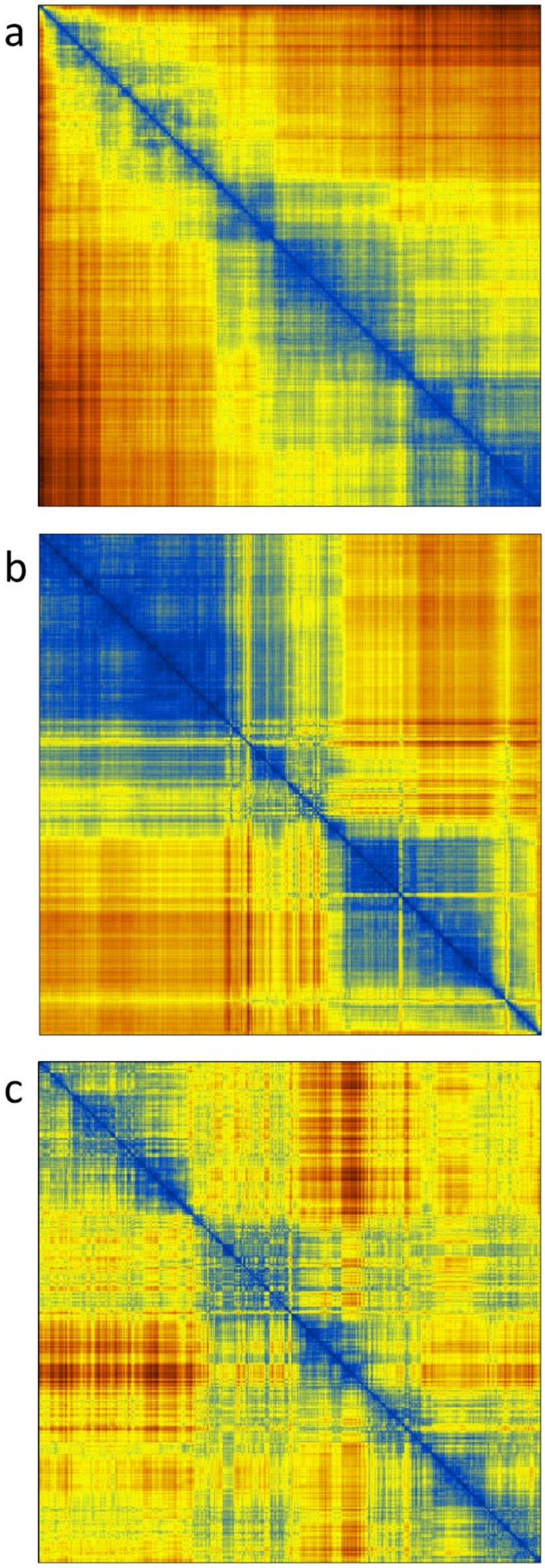


Fig. S3. The 2D RMSD matrix. Panels a) to c) correspond to the antagonist-complex, to the agonist-H3R complex, and to the apo receptor, respectively.

a

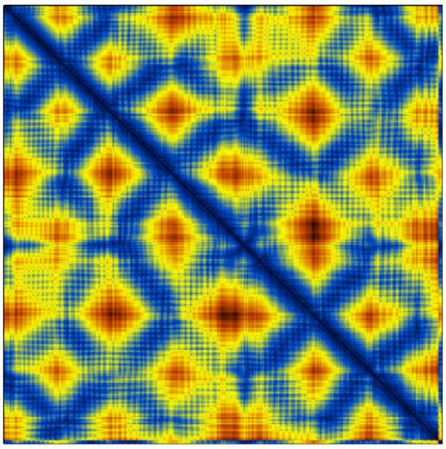

| TM | 1 | 2 | 3 | 4 | 5 | 6 | 7 |
|---|---|---|---|---|---|---|---|
| 1 | | x | | | | | x |
| 2 | | | x | | | x | x |
| 3 | | | | x | x | x | |
| 4 | | | | | | | |
| 5 | | | | | | x | |
| 6 | | | | | | | x |
| 7 | | | | | | | |

b

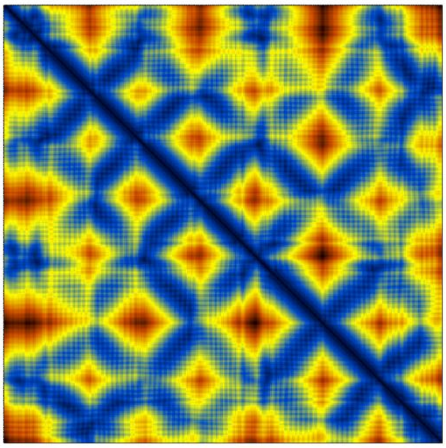

d

| TM | 1 | 2 | 3 | 4 | 5 | 6 | 7 |
|---|---|---|---|---|---|---|---|
| 1 | | x | | | | | x |
| 2 | | | x | x | | | x |
| 3 | | | | x | x | x | x |
| 4 | | | | | x | | |
| 5 | | | | | | x | |
| 6 | | | | | | | x |
| 7 | | | | | | | |

c

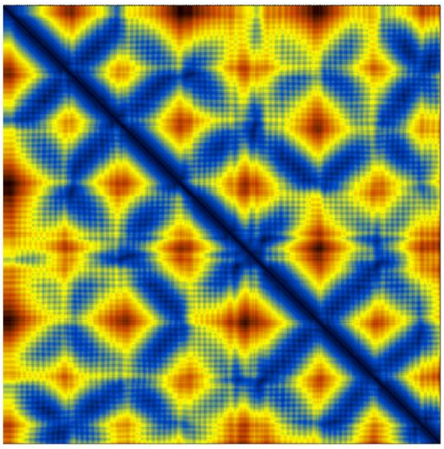

| TM | 1 | 2 | 3 | 4 | 5 | 6 | 7 |
|---|---|---|---|---|---|---|---|
| 1 | | x | | | | | x |
| 2 | | | x | x | | | x |
| 3 | | | | x | x | x | x |
| 4 | | | | | x | | |
| 5 | | | | | | x | |
| 6 | | | | | | | x |
| 7 | | | | | | | |

Fig. S4. The overall production stage 2D contact maps. Panels a) to c) correspond to the antagonist- and agonist-H3R complexes, and to the apo receptor, respectively. Panel d) Relative positions of the TM helices in the form of a symmetric matrix as derived from panels a to c. A proximity between TM helices is represented by the presence of a matrix element.

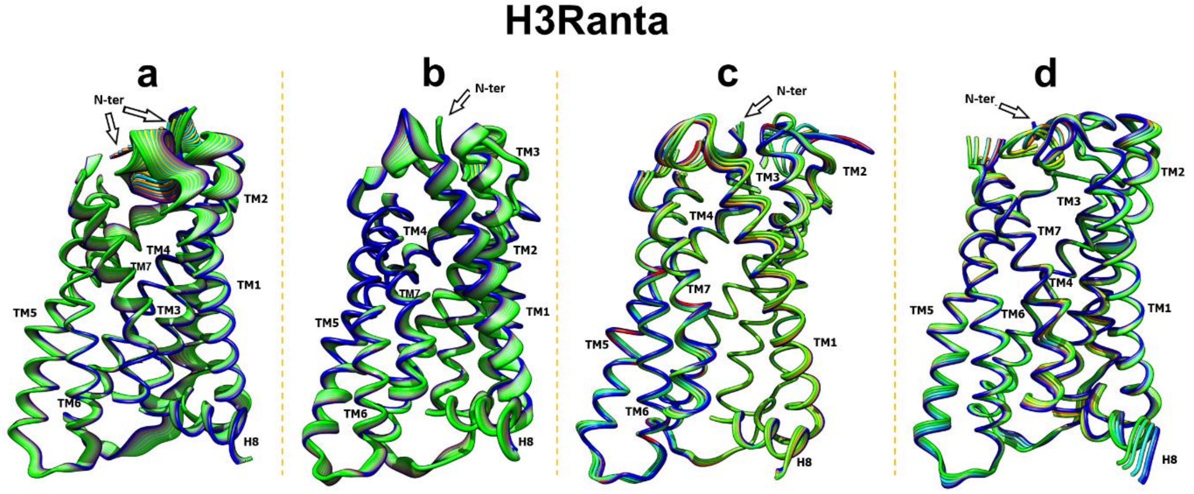


Fig. S5. Clustering of the antagonist state structures. Visualization of the movements of the first principal component PC1 in each of the four clusters, from green to blue, depicting low to high atomic displacements. Panels a) cluster C1, b) cluster C2, c) cluster C3, and d) cluster C4 as determined with the Carma package.

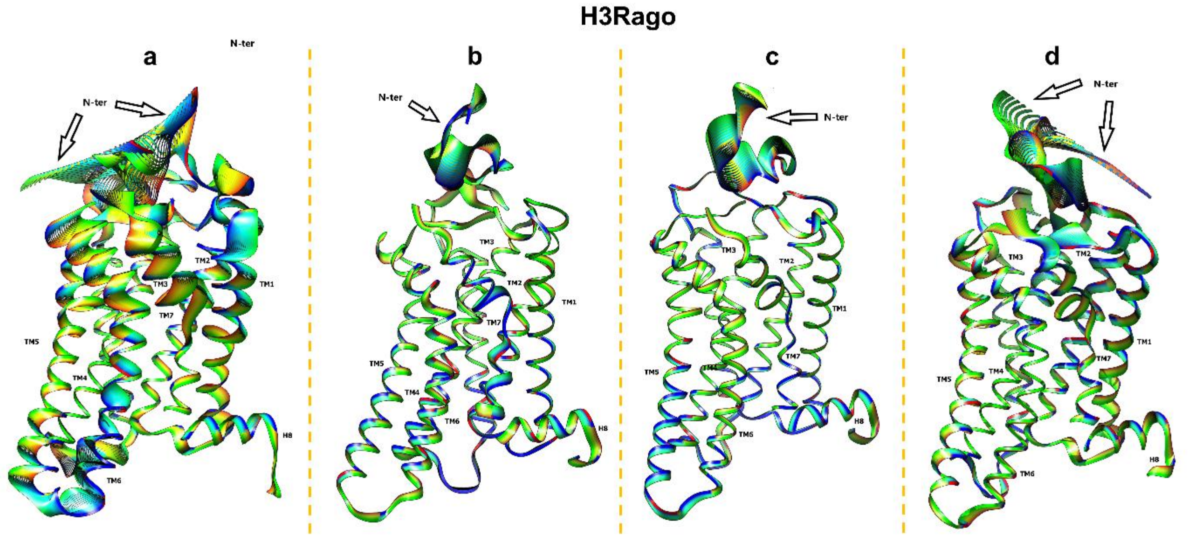


Fig. S6. Clustering of the agonist state structures for section 1. Visualization of the movements of the first principal component PC1 in the four clusters. Color scale as in Fig. S5. Panels a) cluster C1, b) cluster C2, c) cluster C3, and d) cluster C4 as determined with the Carma package.

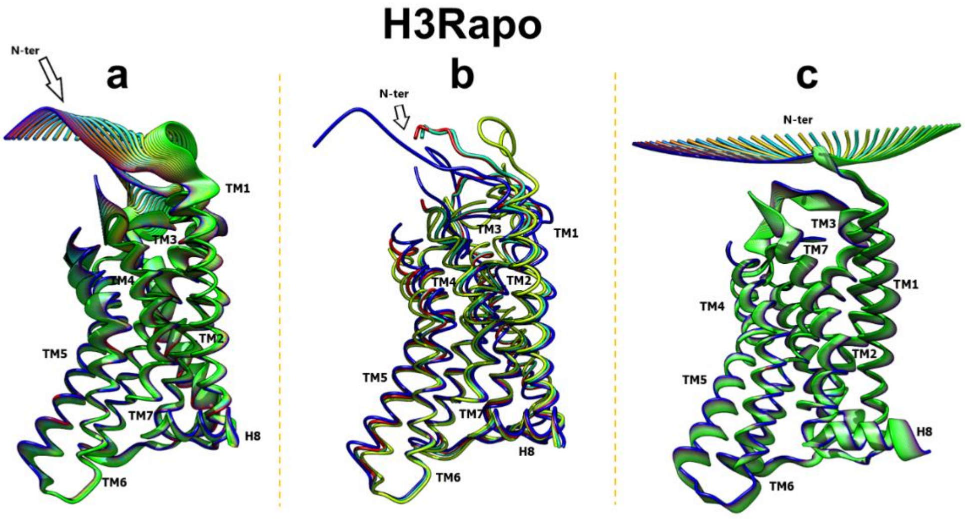


Fig. S7. Clustering of the apo state structure. Visualization of the movements of the first principal component PC1 of the three clusters C1, C2 and C3 as determined by the Carma package. Color scale as in Fig. S5. Panels, a) cluster C1, b) cluster C2, and c) cluster C3.

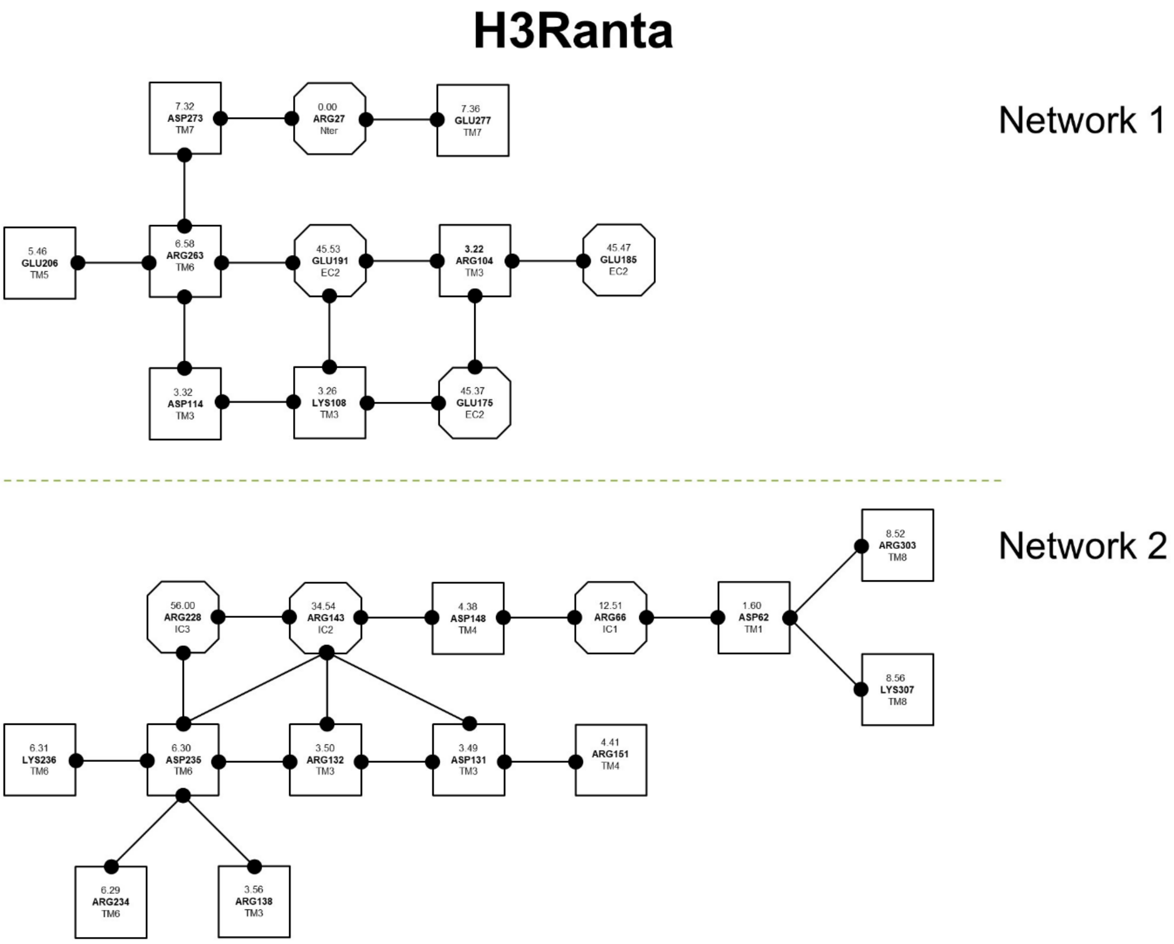

Fig. S8. Networks of charged residues for the antagonist-H3R complex. The two charged amino acids networks stabilize the structure of the receptor, important for the formation of an electrostatic clamp. The boxes correspond to the TM residues and the octagons to N-ter and loop residues.

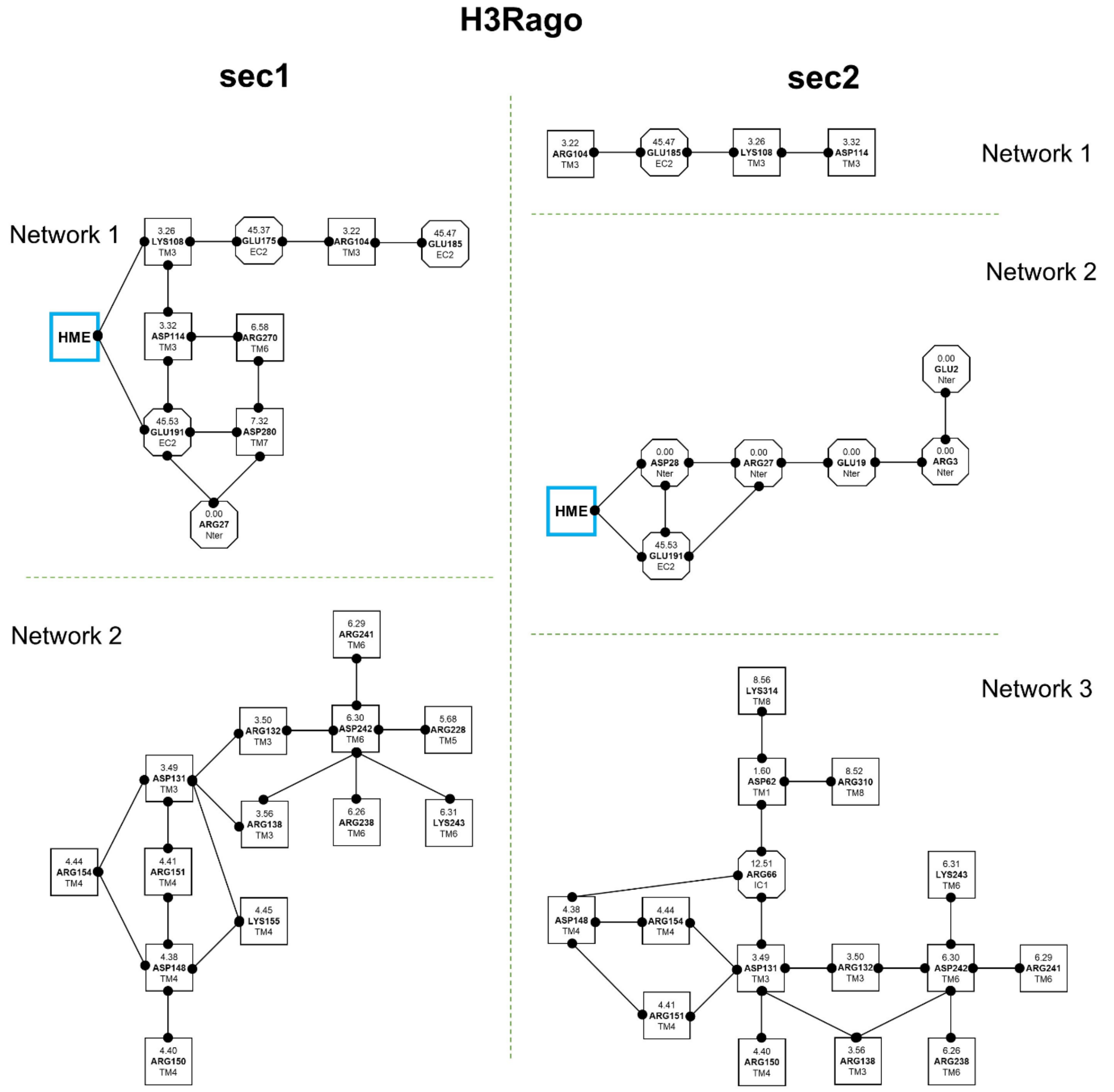


Fig. S9. Networks of charged residues for the agonist-H3R complex. In the two first sections at least one cluster interacts with the ligand. Section 1 in the trajectory shows two inner electrostatic networks, whereas the second section shows three. Shapes as in Fig. S8.

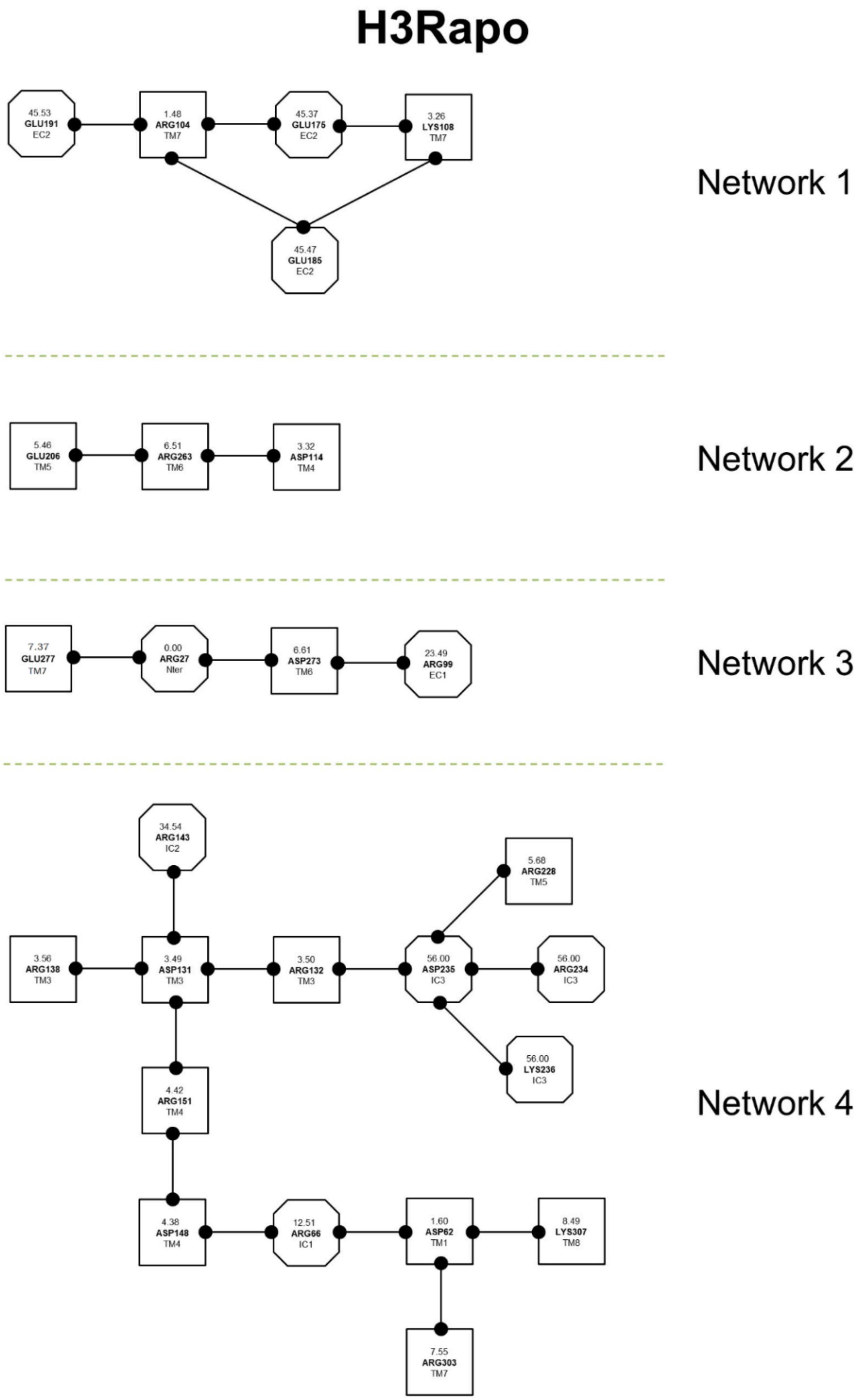


Fig. S10. Networks of charged residues for the apo receptor. The four charged amino acids networks stabilize the apo structure. The first three networks involve less than six residues. Shapes as in Fig. S8.

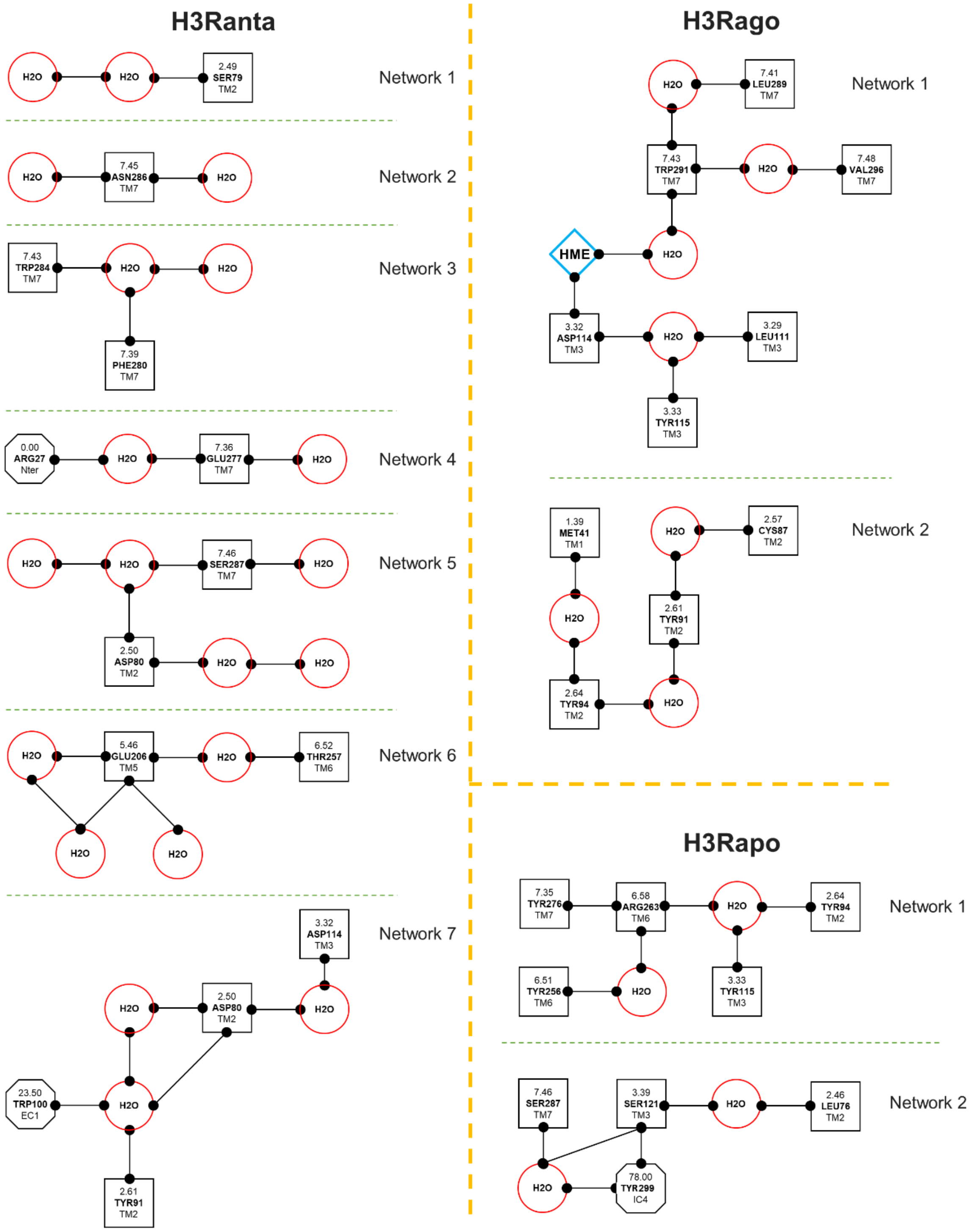


Fig. S11. H-bonded networks. a) Antagonist-H3R complex. The seven networks involve several internal water molecules and clusters, but not the CPX. b) Agonist-H3R complex. The first network shows a water molecule mediating the interaction between the HSM ligand and Trp7.43. c) Apo receptor. In all three systems, all but one are water-mediated interactions.

Many of these interactions are polyvalent. The boxes correspond to the TM residues, the red circles to water molecules and the octagons to N-ter and loop residues.

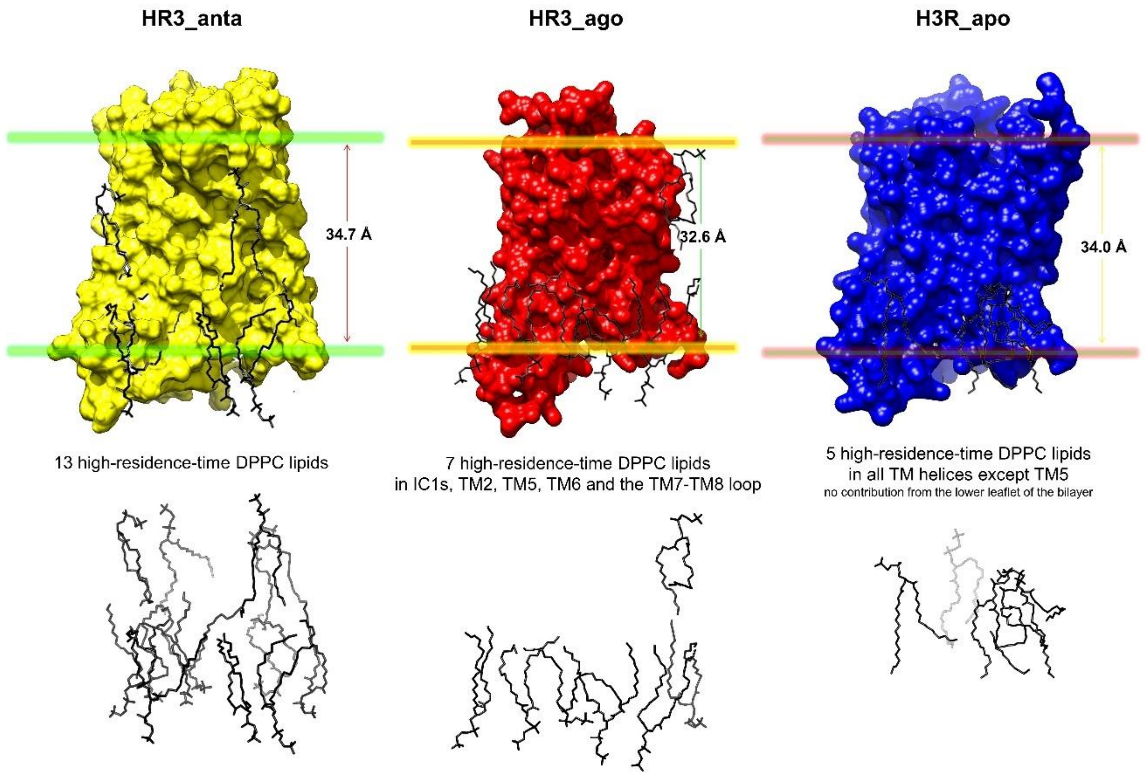


Fig. S12. Lipid-H3R interactions for the three systems. The images are representatives from the whole production phase of the simulation. The upper part shows the thickness of the lipid layer; the lower part view shows just the lipids without the H3R and allows to see the lipids in the backward part of the membrane.

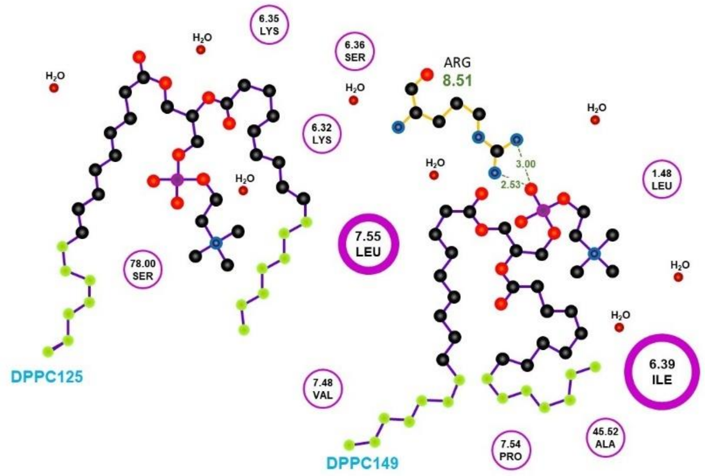


Fig. S13. 2D-binding major mode of DPPC lipids 125 and 149 to the H3 receptor in the antagonist state showing the interacting amino acid residues.

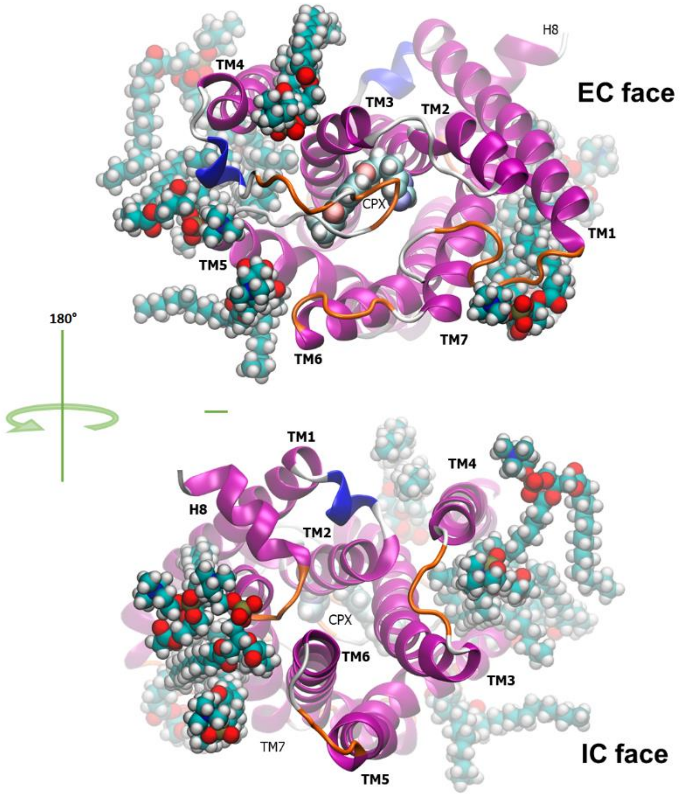


Fig. S14. 3D representation of the average binding mode of DPPC lipids to the H3 receptor in the antagonist state. Above, the extracellular face; below, the intracellular face. The lipids and the CPX ligand are visualized in CPK code.

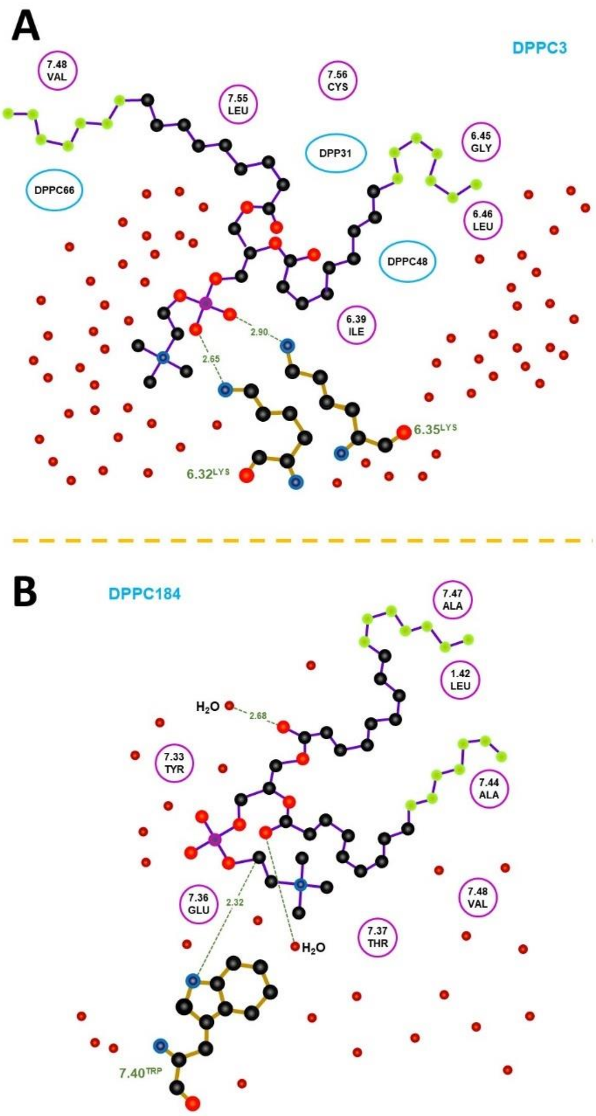


Fig. S15. 2D-binding major mode of DPPC lipids to the H3 receptor in the a) agonist, and b) apo state showing the interaction of amino acid residues and lipids. Multiple DPPC interactions can be observed. Several water molecules (in red) are present.

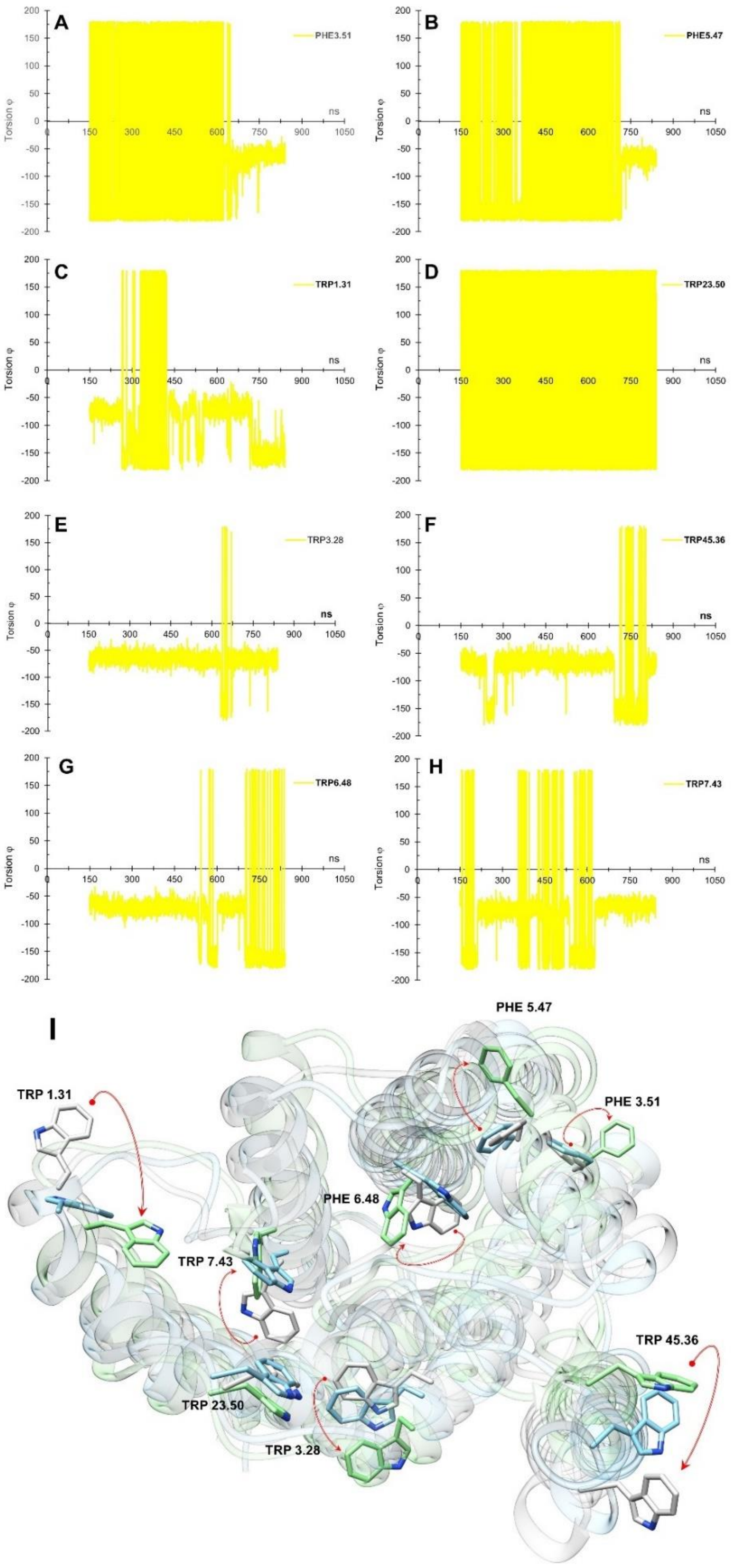


Fig. S16. χ1 torsion angle time evolution for the side chain of selected residues of the antagonist complex.

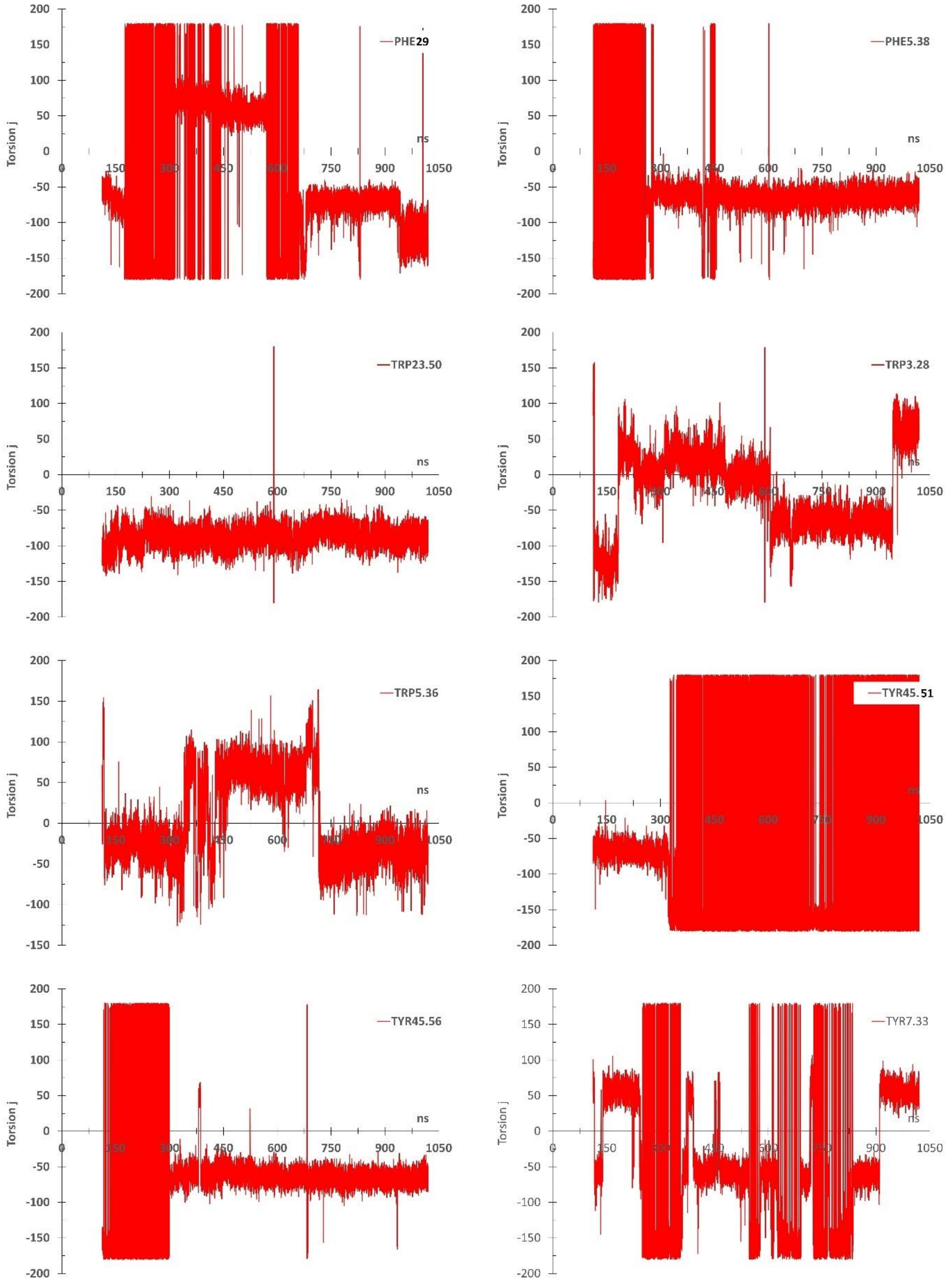


Fig. S17. χ1 torsion angle time evolution for the side chain of selected residues of the agonist complex.

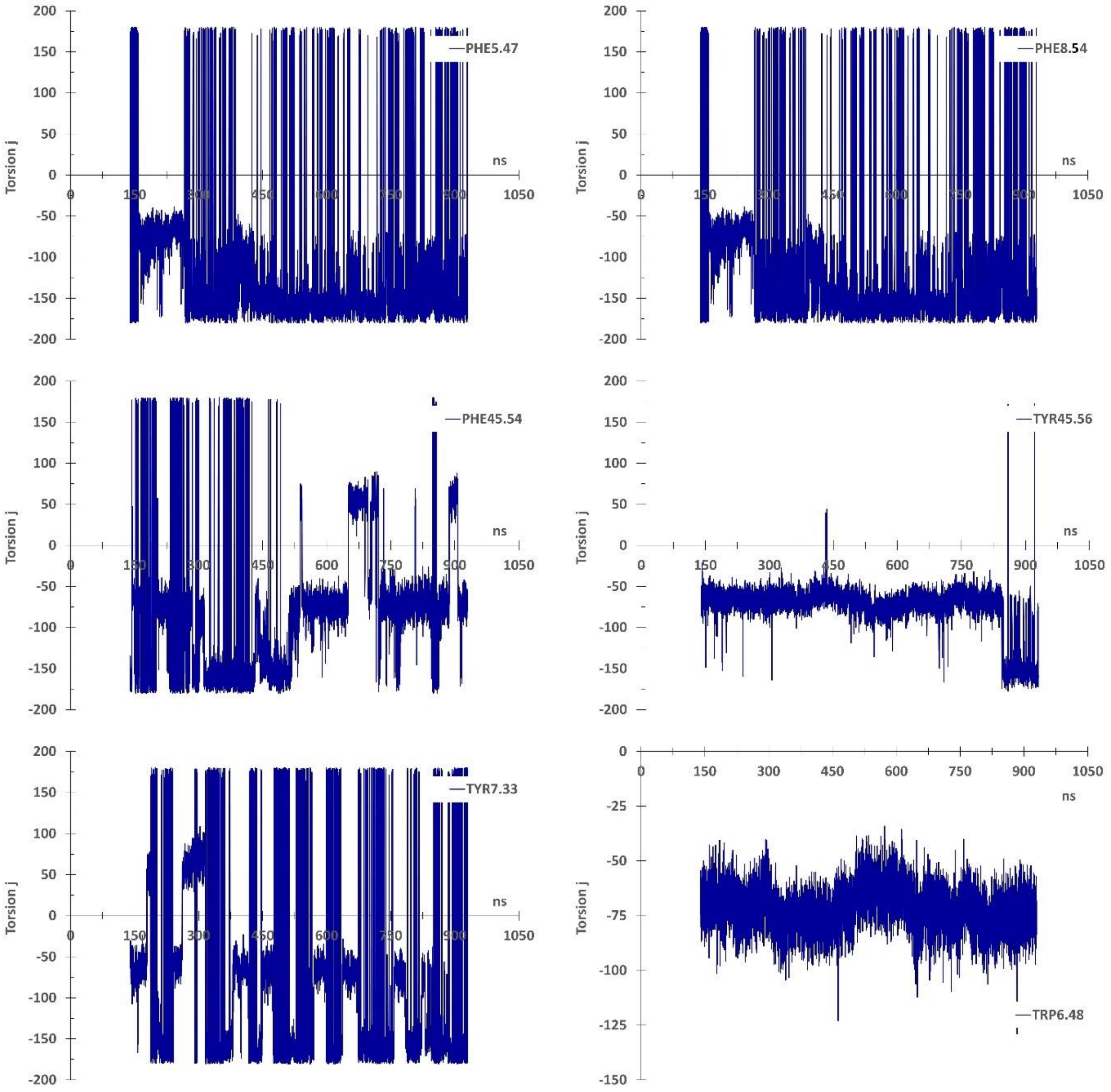


Fig. S18. $\chi_1$ torsion angle time evolution for the side chain of selected residues of the apo receptor.

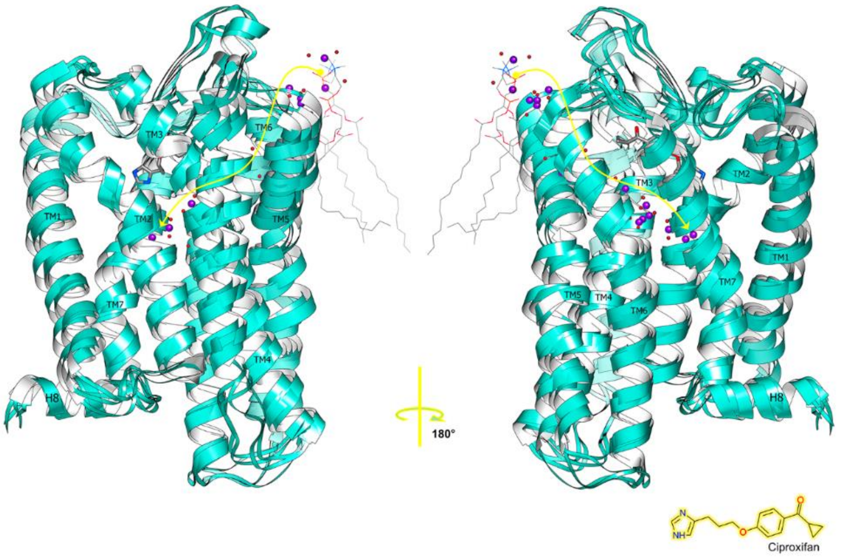


Fig. S19. Schematic illustration of the pathway taken by a $K^+$ metal ion (purple spheres) when penetrating the receptor from the extra-cytoplasmic space and binding to the $Na^+$ allosteric site. The curve in yellow denotes the binding pathway. Red spheres are water molecules and wire lines represent DPPC molecules.

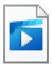

Fig. S20.avi

Fig. S20. Video sequence of the spontaneous incorporation of the $K^+$ ion into the orthosteric site. (As a separate file).

TABLES

| | H3Ranta | | | | | | | | |
|---|---|---|---|---|---|---|---|---|---|
| | Hbond | | | | residencetime | Hbond | | | residencetime |
| | ARG104 | 3.22 | GLU175 | 45.37 | 100.00% | GLU191 | 45.53 | CPX | 47.37% |
| | ARG143 | 34.54 | ASP131 | 3.49 | 100.00% | PHE192 | 45.54 | CPX | 46.10% |
| | GLY186 | 45.48 | GLU191 | 45.32 | 100.00% | | | | |
| | ARG27 | 0.00 | GLU277 | 7.36 | 96.38% | | | | |
| * | THR278 | 7.37 | TYR274 | 7.33 | 91.76% | | | | |
| * | THR306 | 8.55 | ARG302 | 8.51 | 88.02% | | | | |
| * | ILE200 | 5.40 | TRP196 | 5.36 | 87.96% | | | | |
| * | THR201 | 5.41 | TYR197 | 5.37 | 78.94% | | | | |
| | ARG303 | 8.52 | ASP62 | 1.60 | 73.81% | | | | |
| | GLU185 | 45.47 | GLU191 | 45.53 | 73.63% | | | | |
| | TYR189 | 45.51 | GLU191 | 45.53 | 73.59% | | | | |
| * | PHE305 | 8.54 | PHE301 | 8.50 | 72.93% | | | | |
| | HSD298 | 78.00 | SER241 | 6.36 | 72.71% | | | | |
| * | MET41 | 1.39 | LEU37 | 1.35 | 71.89% | | | | |

| | H3Rago-sec1 | | | | | | | | |
|---|---|---|---|---|---|---|---|---|---|
| | Hbond | | | | residencetime | Hbond | | | residencetime |
| | ARG27 | 0.00 | GLU191 | 45.32 | 100.00% | HME | ASP114 | 3.32 | 48.07% |
| | ARG310 | 8.52 | ASP62 | 1.60 | 98.50% | | | | |
| * | SER254 | 6.42 | ALA250 | 6.38 | 84.78% | | | | |
| | TRP174 | 45.36 | PRO169 | 4.59 | 81.34% | | | | |
| | ARG310 | 8.52 | TYR306 | 78.00 | 81.32% | | | | |
| | LYS108 | 3.26 | GLU175 | 45.37 | 77.41% | | | | |
| * | ASP80 | 2.50 | LEU76 | 2.46 | 72.77% | | | | |
| * | LYS247 | 6.34 | LYS243 | 6.31 | 71.46% | | | | |
| * | PHE312 | 8.54 | PHE308 | 8.50 | 70.75% | | | | |
| * | THR313 | 8.55 | ARG309 | 8.51 | 70.16% | | | | |

| H3Rago-sec2 | | | | | | | | |
|---|---|---|---|---|---|---|---|---|
| ARG3 | 0.00 | GLU19 | 0.00 | 100.00% | HME | ASP280 | 7.32 | 44.37% |
| ARG310 | 8.52 | TYR306 | 78.00 | 100.00% | | | | |
| PHE256 | 6.44 | ILE252 | 6.40 | 99.48% | | | | |
| SER254 | 6.42 | ALA250 | 6.38 | 93.59% | | | | |
| LYS247 | 6.35 | LYS243 | 6.31 | 90.47% | | | | |
| TRP174 | 45.36 | PRO169 | 4.59 | 89.43% | | | | |
| LEU239 | 6.27 | GLN235 | 56.00 | 88.39% | | | | |
| ALA157 | 4.47 | VAL153 | 4.43 | 85.27% | | | | |
| ARG27 | 0.00 | GLU191 | 45.53 | 83.54% | | | | |
| SER30 | 0.00 | GLU284 | 7.36 | 83.19% | | | | |
| ARG228 | 5.68 | GLN235 | 56.00 | 75.91% | | | | |
| ARG104 | 3.22 | GLU185 | 45.47 | 74.87% | | | | |
| PHE86 | 2.56 | LEU82 | 2.52 | 74.52% | | | | |
| THR229 | 5.69 | ILE225 | 5.65 | 74.52% | | | | |
| ASP80 | 2.50 | LEU76 | 2.46 | 74.18% | | | | |
| PHE312 | 8.54 | PHE308 | 8.50 | 73.83% | | | | |
| LYS108 | 3.26 | GLU175 | 45.37 | 71.06% | | | | |
| ALA250 | 6.38 | ALA246 | 6.34 | 70.36% | | | | |

| H3Rago-sec3 | | | | |
|---|---|---|---|---|
| SER30 | 0.00 | GLU284 | 7.36 | 100.00% |
| SER254 | 6.42 | ALA250 | 6.38 | 99.39% |
| LYS247 | 6.35 | LYS243 | 6.31 | 98.99% |
| PHE256 | 6.44 | ILE252 | 6.40 | 96.96% |
| LEU239 | 6.27 | GLN235 | 56.00 | 95.33% |
| ARG310 | 8.52 | TYR306 | 78.00 | 91.68% |
| SER248 | 6.36 | LYS244 | 6.32 | 87.63% |
| TRP174 | 45.36 | PRO169 | 4.59 | 86.41% |
| ARG27 | 0.00 | GLU191 | 45.53 | 85.19% |
| ASP80 | 2.50 | LEU76 | 2.46 | 82.76% |
| THR313 | 8.55 | ARG309 | 8.51 | 80.73% |
| ALA157 | 4.47 | VAL153 | 4.43 | 80.73% |
| TYR91 | 2.61 | CYS87 | 2.57 | 80.32% |
| ARG228 | 5.68 | GLN235 | 56.00 | 80.32% |
| LYS108 | 3.26 | ALA170 | 4.60 | 75.05% |

| H3Rago-sec4 | | | | |
|---|---|---|---|---|
| ARG3 | 0.00 | GLU191 | 45.53 | 100.00% |
| SER254 | 6.42 | ALA250 | 6.38 | 88.07% |
| ASP80 | 2.50 | LEU76 | 2.46 | 81.14% |
| ARG310 | 8.52 | TYR306 | 78.00 | 78.73% |
| TRP174 | 45.36 | PRO169 | 4.59 | 77.59% |
| PHE312 | 3.50 | PHE308 | 8.50 | 75.65% |

| | H3RApo | | | | |
|---|---|---|---|---|---|
| | Hbond | | | | residencetime |
| * | SER247 | 6.42 | ALA243 | 6.38 | 86.97% |
| * | SER79 | 2.49 | ASN75 | 2.45 | 86.51% |
| * | ILE78 | 2.48 | LEU74 | 2.44 | 85.91% |
| * | VAL246 | 6.41 | LEU242 | 6.37 | 81.37% |
| | TRP174 | 45.36 | PRO169 | 4.59 | 78.15% |

Supplementary Table S1. H-bonding residue pairs for residence times greater than 70% for the antagonist, agonist and apo systems, respectively. Includes intra-helical H-bonds (*), except sections 2-4 of the agonist complex. HSD stands for Histidine. Includes main-chain and side-chain H-bonds.

a)

| State | | Number of waters |
|---|---|---|
| H3R ANTA | | 61.34 |
| H3R AGO | | |
| | Section 1 | 35.15 |
| | Section 2 | 19.83 |
| | Section 3 | 19.00 |
| | Section 4 | 24.91 |
| H3R APO | | 48.95 |

b)

**N-ter**

| H3Ranta | | H3Rago | | H3Rapo | |
|---|---|---|---|---|---|

**TM1**

| H3Ranta | | H3Rago | | H3Rapo | |
|---|---|---|---|---|---|
| | | MET41 | 1.39 | | |
| | | LEU44 | 1.42 | | |

**ICL1**

| H3Ranta | | H3Rago | | H3Rapo | |
|---|---|---|---|---|---|

**TM2**

| H3Ranta | | H3Rago | | H3Rapo | |
|---|---|---|---|---|---|
| | | ASN70 | 2.4 | | |
| SER79 | 2.49 | | | SER79 | 2.49 |
| ASP80 | 2.5 | | | | |
| PHE81 | 2.51 | | | | |
| VAL83 | 2.53 | VAL83 | 2.53 | VAL83 | 2.53 |
| | | CYS87 | 2.57 | | |
| ILE88 | 2.58 | ILE88 | 2.58 | ILE88 | ILE88 |
| | | LEU90 | 2.6 | | |
| TYR91 | 2.61 | TYR91 | 2.61 | TYR91 | TYR91 |
| TYR94 | 2.64 | TYR94 | 2.64 | | |

**ECL1**

| H3Ranta | | H3Rago | | H3Rapo | |
|---|---|---|---|---|---|

**TM3**

| H3Ranta | | H3Rago | | H3Rapo | |
|---|---|---|---|---|---|
| CYS107 | 3.25 | CYS107 | 3.25 | | |
| TRP110 | 3.28 | | | | |
| LEU111 | 3.29 | LEU111 | 3.29 | LEU111 | 3.29 |
| ASP114 | 3.32 | ASP114 | 3.32 | ASP114 | 3.32 |
| TYR115 | 3.33 | TYR115 | 3.33 | TYR115 | 3.33 |
| LEU117 | 3.35 | | | LEU117 | 3.35 |
| CYS118 | 3.36 | | | | |
| SER121 | 3.39 | | | SER121 | 3.39 |
| VAL122 | 3.4 | | | VAL122 | 3.4 |

**ICL2**

| H3Ranta | | H3Rago | | H3Rapo | |
|---|---|---|---|---|---|

**TM4**

| H3Ranta | | H3Rago | | H3Rapo | |
|---|---|---|---|---|---|

**ECL2**

| H3Ranta | | H3Rago | | H3Rapo | |
|---|---|---|---|---|---|
| | | GLU175 | 45.37 | | |
| | | TYR189 | 45.51 | | |
| | | ALA190 | 45.52 | | |
| GLU191 | 45.53 | GLU191 | 45.53 | | |
| PHE192 | 45.54 | PHE192 | 45.54 | PHE192 | 45.54 |
| PHE193 | 45.55 | | | | |
| TYR194 | 45.56 | | | TYR194 | 45.56 |

**TM5**

| H3Ranta | | H3Rago | | H3Rapo | |
|---|---|---|---|---|---|
| | | PHE198 | 5.38 | | |
| LEU199 | 5.39 | | | LEU199 | 5.39 |
| ALA202 | 5.42 | | | | |
| SER203 | 5.43 | | | | |
| GLU206 | 5.46 | | | | |
| PHE207 | 5.47 | | | PHE207 | 5.47 |

**ICL3**

| H3Ranta | | H3Rago | | H3Rapo | |
|---|---|---|---|---|---|

**TM6**

| H3Ranta | | H3Rago | | H3Rapo | |
|---|---|---|---|---|---|
| | | LEU249 | 6.37 | | |
| TRP253 | 6.48 | | | | |
| TYR256 | 6.51 | | | TYR256 | 6.51 |
| MET260 | 6.55 | | | MET260 | 6.55 |
| ARG263 | 6.58 | TYR263 | 6.51 | ARG263 | 6.58 |

**ECL3**

| H3Ranta | | H3Rago | | H3Rapo | |
|---|---|---|---|---|---|

**TM7**

| H3Ranta | | H3Rago | | H3Rapo | |
|---|---|---|---|---|---|
| TYR276 | 7.35 | TYR283 | 7.35 | TYR276 | 7.35 |
| GLU277 | 7.36 | GLU284 | 7.36 | | |
| SER279 | 7.38 | | | | |
| PHE280 | 7.39 | PHE287 | 7.39 | PHE280 | 7.39 |
| LEU283 | 2.42 | | | LEU283 | 2.42 |
| TRP284 | 7.43 | TRP291 | 7.43 | TRP284 | 7.43 |
| ASN286 | 7.45 | | | ASN286 | 7.45 |
| SER287 | 7.46 | | | SER287 | 7.46 |
| ASN290 | 7.49 | SER294 | 7.49 | ASN290 | 7.49 |

**ICL4**

| H3Ranta | | H3Rago | | H3Rapo | |
|---|---|---|---|---|---|

**TM68**

| H3Ranta | | H3Rago | | H3Rapo | |
|---|---|---|---|---|---|

**C-ter**

| H3Ranta | | H3Rago | | H3Rapo | |
|---|---|---|---|---|---|

Supplementary Table S2. a) Average water population in the internal cavity; b) Amino acid residues involved in the formation of the receptor cavity for the antagonist, agonist and apo systems.

## H3Ranta

| Cluster | 1 | |
|---|---|---|
| TM1 | LEU54 | 1.52 |
| TM1 | ALA58 | 1.56 |
| TM1/TM2 | LEU65 | 12.50 |

| Cluster | 2 | |
|---|---|---|
| Nter/TM1 | ALA23 | 0.00 |
| TM2 | PHE86 | 2.56 |
| TM2 | CYS87 | 2.57 |
| TM2 | LEU90 | 2.60 |
| TM2/TM3 | TRP100 | 23.50 |
| TM3 | LEU106 | 3.24 |
| TM3 | TRP110 | 3.28 |

| Cluster | 3 | |
|---|---|---|
| TM2 | LEU82 | 2.52 |
| TM2 | VAL83 | 2.53 |
| TM3 | VAL113 | 3.31 |
| TM3 | LEU117 | 3.35 |

| Cluster | 4 | |
|---|---|---|
| TM2 | LEU73 | 2.43 |
| TM3 | ILE128 | 3.46 |
| TM5 | ILE221 | 5.61 |
| TM6 | LEU242 | 6.37 |
| TM6 | 2ILE245 | 6.40 |
| TM7 | CYS297 | 7.56 |

| Cluster | 5 | |
|---|---|---|
| TM3 | LEU109 | 3.27 |
| TM3 | VAL112 | 3.30 |
| TM4 | LEU166 | 4.56 |

| Cluster | 6 | |
|---|---|---|
| TM6 | ALA254 | 6.49 |
| TM6 | PRO255 | 6.50 |
| TM6 | ILE262 | 6.57 |
| TM6/TM7 | VAL271 | 67.00 |

## H3Rago

### sec1

| Cluster | 1 | |
|---|---|---|
| TM1 | VAL49 | 1.47 |
| TM1 | LEU54 | 1.47 |
| TM1 | MET56 | 1.54 |
| TM1 | LEU57 | 1.55 |
| TM1 | ALA58 | 1.56 |
| TM1 | PHE59 | 1.57 |
| TM1 | ALA61 | 1.59 |
| TM1/TM2 | LEU65 | 12.50 |
| TM7 | PRO302 | 7.54 |

| Cluster | 2 | |
|---|---|---|
| Nter/TM1 | ALA26 | 0.00 |
| TM2/TM3 | TRP100 | 23.50 |
| TM3 | CYS107 | 3.25 |

| Cluster | 3 | |
|---|---|---|
| TM2 | LEU76 | 2.46 |
| TM3 | ILE125 | 3.43 |
| TM5 | VAL214 | 5.54 |
| TM6 | LEU249 | 6.37 |

| Cluster | 4 | |
|---|---|---|
| TM3 | PHE133 | 3.51 |
| TM3 | VAL136 | 3.54 |
| TM5 | PHE216 | 5.56 |

| Cluster | 5 | |
|---|---|---|
| TM3 | VAL112 | 3.30 |
| TM3 | LEU116 | 3.34 |
| TM3 | ALA119 | 3.37 |
| TM4 | LEU166 | 4.56 |
| TM5 | ALA202 | 5.42 |

| Cluster | 6 | |
|---|---|---|
| TM1 | LEU44 | 1.42 |
| TM3 | VAL122 | 3.40 |
| TM3 | VAL126 | 3.44 |
| TM3 | ILE128 | 3.46 |
| TM5 | PHE207 | 5.47 |
| TM5 | PHE211 | 5.51 |
| TM6 | ILE255 | 6.43 |

### sec2

| Cluster | 1 | |
|---|---|---|
| TM1 | VAL49 | 1.47 |
| TM1 | LEU54 | 1.52 |
| TM1 | MET56 | 1.54 |
| TM1 | LEU57 | 1.55 |
| TM1 | ALA58 | 1.56 |
| TM1 | PHE59 | 1.57 |
| TM1 | ALA61 | 1.59 |
| TM1/TM2 | LEU65 | 12.50 |

| Cluster | 2 | |
|---|---|---|
| TM2 | LEU82 | 2.53 |
| TM2 | PHE86 | 2.56 |
| TM3 | VAL112 | 3.30 |

| Cluster | 3 | |
|---|---|---|
| TM3 | ALA119 | 3.37 |
| TM/TM5 | LEU177 | 45.39 |
| TM4/TM5 | PHE192 | 45.54 |
| TM5 | PHE193 | 45.55 |
| TM5 | ALA202 | 5.42 |
| TM6 | LEU266 | 6.54 |

| Cluster | 4 | |
|---|---|---|
| TM3 | CYS118 | 3.36 |
| TM3 | VAL122 | 3.40 |
| TM5 | PHE207 | 5.47 |
| TM5 | PHE211 | 5.51 |
| TM6 | ILE255 | 6.43 |
| TM6 | PHE256 | 6.44 |

| Cluster | 5 | |
|---|---|---|
| TM3 | ILE125 | 3.43 |
| TM3 | 1VAL126 | 3.44 |
| TM5 | VAL214 | 5.54 |
| TM5 | PHE216 | 5.56 |
| TM6 | LEU249 | 6.37 |
| TM6 | ILE252 | 6.40 |
| TM7 | CYS304 | 7.56 |

| Cluster | 6 | |
|---|---|---|
| TM6 | LEU265 | 6.53 |
| TM6 | ILE269 | 6.57 |
| TM6/TM7 | VAL278 | 76.00 |
| TM6/TM7 | VAL279 | 7.31 |

### sec3

| Cluster | 1 | |
|---|---|---|
| TM1 | LEU54 | 1.52 |
| TM1 | LEU57 | 1.55 |
| TM1 | ALA58 | 1.56 |
| TM1 | PHE59 | 1.57 |
| TM1 | ALA61 | 1.59 |
| TM1/TM2 | LEU65 | 12.5 |
| TM7 | PRO302 | 7.54 |

| Cluster | 2 | |
|---|---|---|
| TM2 | LEU82 | 2.52 |
| TM2 | PHE86 | 2.56 |
| TM3 | VAL112 | 3.30 |

| Cluster | 3 | |
|---|---|---|
| TM3 | LEU111 | 3.29 |
| TM3 | ALA119 | 3.37 |
| TM4/TM5 | PHE192 | 45.54 |
| TM5 | ILE200 | 5.40 |
| TM5 | ALA202 | 5.42 |
| TM6 | LEU266 | 6.54 |

| Cluster | 4 | |
|---|---|---|
| TM3 | ILE125 | 3.43 |
| TM2 | LEU76 | 2.46 |
| TM5 | VAL214 | 5.54 |
| TM3 | ILE128 | 3.46 |

| Cluster | 5 | |
|---|---|---|
| TM2 | PHE71 | 2.41 |
| TM2 | PHE72 | 2.42 |
| TM2 | LEU73 | 2.43 |
| TM3 | PHE123 | 3.41 |
| TM3 | LEU127 | 3.45 |
| TM4 | LEU158 | 4.48 |

| Cluster | 6 | |
|---|---|---|
| TM3 | VAL122 | 3.40 |
| TM3 | VAL126 | 3.44 |
| TM5 | PHE207 | 5.47 |
| TM5 | PHE208 | 5.48 |
| TM5 | PRO210 | 5.50 |
| TM5 | PHE211 | 5.51 |

### sec4

| Cluster | 1 | |
|---|---|---|
| Nter/TM1 | ALA23 | 0.00 |
| Nter/TM1 | ALA26 | 0.00 |
| Nter/TM1 | PHE29 | 0.00 |
| Nter/TM1 | ALA31 | 1.29 |

| Cluster | 2 | |
|---|---|---|
| TM1 | LEU54 | 1.52 |
| TM1 | LEU57 | 1.55 |
| TM1 | ALA58 | 1.56 |
| TM1 | PHE59 | 1.57 |
| TM1/TM2 | LEU65 | 12.50 |
| TM7 | PRO302 | 7.54 |

| Cluster | 3 | |
|---|---|---|
| TM2 | LEU90 | 2.60 |
| TM2/TM3 | TRP100 | 23.50 |
| TM2/TM3 | PHE102 | 23.52 |
| TM3 | CYS107 | 3.25 |

| Cluster | 4 | |
|---|---|---|
| TM2 | LEU76 | 2.46 |
| TM3 | VAL122 | 3.40 |
| TM3 | PHE123 | 3.41 |
| TM3 | ILE125 | 3.43 |
| TM3 | VAL126 | 3.44 |
| TM3 | ILE128 | 3.46 |
| TM5 | PHE207 | 5.47 |
| TM5 | PHE211 | 5.51 |
| TM5 | VAL214 | 5.54 |
| TM6 | LEU249 | 6.37 |

| Cluster | 5 | |
|---|---|---|
| TM3 | LEU111 | 3.29 |
| TM3 | VAL112 | 3.30 |
| TM3 | LEU116 | 3.34 |
| TM4 | ALA163 | 4.53 |

| Cluster | 6 | |
|---|---|---|
| TM3 | ALA119 | 3.37 |
| TM4 | LEU166 | 4.56 |
| TM5 | ALA202 | 5.42 |

| Cluster | 7 | |
|---|---|---|
| TM3 | VAL136 | 3.54 |
| TM5 | ILE221 | 5.61 |
| TM5 | ILE225 | 5.65 |

| Cluster | 8 | |
|---|---|---|
| TM6 | LEU265 | 6.53 |
| TM6 | ILE269 | 6.57 |
| TM6 | ALA272 | 6.60 |

## H3Rapo

| Cluster | 1 | |
|---|---|---|
| TM1 | MET41 | 1.39 |
| TM1 | ILE45 | 1.43 |
| TM2 | LEU82 | 2.52 |
| TM2 | VAL83 | 2.53 |
| TM3 | VAL113 | 3.31 |
| TM6 | PHE249 | 6.44 |
| TM6 | ILE248 | 6.43 |
| TM7 | PHE280 | 7.39 |
| TM7 | TRP281 | 7.40 |
| TM7 | LEU283 | 7.42 |
| TM7 | VAL289 | 7.48 |

| Cluster | 2 | |
|---|---|---|
| TM1 | VAL49 | 1.47 |
| TM1 | VAL55 | 1.53 |
| TM1 | MET56 | 1.54 |
| TM1 | ALA58 | 1.56 |
| TM1 | PHE59 | 1.57 |
| TM1/MT2 | SER64 | 12.49 |
| TM1/TM2 | LEU65 | 12.50 |
| TM7 | CYS297 | 7.56 |

| Cluster | 3 | |
|---|---|---|
| TM2 | PHE86 | 2.56 |
| TM2 | CYS87 | 2.57 |
| TM2 | LEU90 | 2.60 |
| TM1/TM2 | TRP100 | 23.50 |
| TM1/TM2 | PHE102 | 23.52 |
| TM3 | CYS107 | 3.25 |

| Cluster | 4 | |
|---|---|---|
| TM3 | VAL122 | 3.40 |
| TM3 | 1VAL126 | 3.44 |
| TM5 | PRO210 | 5.50 |
| TM5 | VAL214 | 5.54 |

| Cluster | 5 | |
|---|---|---|
| TM1 | LEU54 | 1.52 |
| TM1 | LEU57 | 1.55 |
| TM7 | PRO295 | 7.54 |

Supplementary Table S3. Hydrophobic clusters at 90% occupancy formed by at least three side chains for the antagonist-H3R complex, the agonist-H3R complex and the apo receptor, respectively.

**HR3anta**

| | | | Residence time | | | Residence time | | | Residence time | | | Residence time | | | Residence time |
|---|---|---|---|---|---|---|---|---|---|---|---|---|---|---|---|
| DPPC | 125 | | 99.98% | 110 | | 99.96% | 161 | | 99.94% | 79 | | 99.78% | 33 | | 98.64% |
| Amino acid residue | LEU296 | 7.55 | 98.24% | PHE123 | 3.41 | 97.55% | LEU158 | 4.48 | 99.43% | PHE29 | 0.00 | 99.16% | TYR197 | 5.37 | 93.97% |
| | ILE244 | 6.39 | 98.09% | LEU205 | 5.45 | 96.59% | ARG154 | 4.44 | 99.04% | TRP281 | 7.40 | 97.01% | ASN195 | 45.57 | 89.37% |
| | LYS240 | 6.35 | 92.74% | LEU127 | 3.45 | 95.87% | LYS155 | 4.45 | 92.68% | TYR274 | 7.33 | 94.92% | PHE198 | 5.38 | 87.40% |
| | CYS297 | 7.56 | 89.91% | TYR130 | 3.48 | 94.89% | PHE123 | 3.41 | 92.10% | THR278 | 7.37 | 89.16% | TRP196 | 5.36 | 74.68% |
| | LYS237 | 6.32 | 88.37% | VAL126 | 3.44 | 85.24% | ARG151 | 4.41 | 89.66% | GLY28 | 0.00 | 78.49% | LEU165 | 4.55 | 72.77% |
| | HIS298 | 78.00 | 87.19% | LEU134 | 3.52 | 85.11% | ARG150 | 4.40 | 86.99% | SER30 | 0.00 | 77.94% | LEU166 | 4.56 | 56.57% |
| | ILE248 | 6.43 | 86.94% | ARG151 | 4.41 | 75.69% | ALA119 | 3.37 | 84.45% | ALA285 | 7.44 | 77.87% | LEU162 | 4.52 | 52.38% |
| | SER241 | 6.36 | 86.02% | VAL122 | 3.40 | 72.52% | LEU162 | 4.52 | 79.77% | LEU282 | 7.41 | 77.13% | THR201 | 5.41 | 52.16% |
| | LEU293 | 7.52 | 82.05% | ALA119 | 3.37 | 62.11% | SER120 | 3.38 | 77.42% | GLU277 | 7.36 | 73.74% | PRO169 | 4.59 | 51.81% |
| | ARG302 | 8.51 | 79.81% | ARG138 | 3.56 | 58.04% | VAL159 | 4.49 | 76.41% | TRP33 | 1.31 | 73.39% | | | |
| | VAL289 | 7.48 | 76.21% | THR201 | 5.41 | 52.29% | LEU116 | 3.34 | 64.88% | LEU40 | 1.38 | 64.02% | | | |
| | LEU251 | 6.46 | 58.81% | LEU212 | 5.52 | 52.28% | | | | ARG27 | 0.00 | 61.61% | | | |
| | | | | | | | | | | ALA31 | 0.00 | 52.52% | | | |
| | | | | | | | | | | ASP273 | 7.32 | 51.81% | | | |

| | | | Residence time | | | Residence time | | | Residence time | | | Residence time |
|---|---|---|---|---|---|---|---|---|---|---|---|---|
| DPPC | 165 | | 98.13% | 184 | | 85.65% | 168 | | 84.83% | 43 | | 82.97% |
| Amino acid residue | LYS240 | 6.35 | 78.55% | TYR130 | 3.48 | 69.46% | VAL60 | 1.58 | 57.42% | LEU109 | 3.27 | 82.89% |
| | TYR222 | 5.62 | 78.16% | PHE133 | 3.51 | 63.47% | MET56 | 1.54 | 53.52% | TYR167 | 4.57 | 82.23% |
| | ARG231 | 6.26 | 74.41% | LEU212 | 5.52 | 63.44% | LEU57 | 1.55 | 51.13% | GLY105 | 3.23 | 81.49% |
| | LEU251 | 6.46 | 72.63% | THR137 | 3.55 | 62.40% | | | | ALA170 | 4.60 | 77.59% |
| | SER247 | 6.42 | 68.94% | ARG138 | 3.56 | 60.11% | | | | ILE171 | 4.61 | 77.55% |
| | ILE244 | 6.39 | 65.96% | PHE208 | 5.48 | 58.10% | | | | ARG104 | 3.22 | 76.98% |
| | ILE225 | 5.65 | 60.48% | LEU134 | 3.52 | 56.56% | | | | LYS108 | 3.26 | 74.05% |
| | ALA243 | 6.38 | 57.24% | PHE217 | 5.57 | 51.97% | | | | LEU106 | 3.24 | 65.22% |
| | LYS236 | 6.31 | 51.21% | | | | | | | SER173 | 4.63 | 65.11% |
| | GLN226 | 5.66 | 50.87% | | | | | | | TRP160 | 4.50 | 61.88% |
| | ASN218 | 5.58 | 50.20% | | | | | | | PHE164 | 4.54 | 59.13% |
| | | | | | | | | | | ALA163 | 4.53 | 58.24% |
| | | | | | | | | | | VAL113 | 3.31 | 56.30% |
| | | | | | | | | | | GLU175 | 45.37 | 55.41% |

| | | | Residence time | | | Residence time | | | Residence time | | | Residence time |
|---|---|---|---|---|---|---|---|---|---|---|---|---|
| DPPC | 31 | | 82.76% | 149 | | 82.46% | 131 | | 81.48% | 166 | | 81.43% |
| Amino acid residue | TRP196 | 5.36 | 75.41% | VAL292 | 7.51 | 81.81% | PHE216 | 5.56 | 62.39% | VAL153 | 4.43 | 52.00% |
| | ILE200 | 5.40 | 72.68% | PRO291 | 7.50 | 79.11% | THR137 | 3.55 | 61.67% | | | |
| | THR204 | 5.44 | 71.88% | PRO295 | 7.54 | 78.79% | PHE133 | 3.51 | 58.19% | | | |
| | PHE208 | 5.48 | 62.75% | ARG302 | 8.51 | 78.78% | LEU212 | 5.52 | 51.67% | | | |
| | HIS267 | 67.00 | 58.52% | ALA47 | 1.45 | 78.63% | | | | | | |
| | ALA264 | 6.59 | 57.63% | GLY51 | 1.49 | 77.95% | | | | | | |
| | SER203 | 5.43 | 57.39% | LEU54 | 1.52 | 77.17% | | | | | | |
| | PHE207 | 5.47 | 57.37% | PHE305 | 8.54 | 76.24% | | | | | | |
| | LEU199 | 5.39 | 56.78% | LEU296 | 7.55 | 74.25% | | | | | | |
| | | | | ALA288 | 7.47 | 73.66% | | | | | | |
| | | | | THR48 | 1.46 | 70.94% | | | | | | |
| | | | | VAL289 | 7.48 | 70.17% | | | | | | |
| | | | | LEU50 | 1.48 | 67.78% | | | | | | |
| | | | | LEU44 | 1.42 | 52.75% | | | | | | |

Supplementary Table S4 (see below).

**H3Rago**

**sec1**

| | | | Residence time | | | Residence time | | | Residence time | | | Residence time |
|---|---|---|---|---|---|---|---|---|---|---|---|---|
| DPPC | 2 | | 100.00% | 47 | | 98.43% | 183 | | 94.05% | 7 | | 93.16% |
| Amino acid residue | ILE251 | 6.39 | 87.11% | TYR222 | 5.62 | 98.43% | VAL92 | 2.62 | 77.96% | VAL299 | 7.51 | 59.14% |
| | LYS247 | 6.35 | 81.72% | ALA250 | 6.38 | 98.18% | ILE88 | 2.58 | 75.50% | ARG309 | 8.51 | 53.02% |
| | ILE255 | 6.43 | 81.36% | ASN218 | 5.58 | 98.07% | PRO93 | 2.63 | 75.20% | THR313 | 8.55 | 52.78% |
| | LEU303 | 7.55 | 77.37% | LYS247 | 6.35 | 95.25% | LEU96 | 2.66 | 72.26% | | | |
| | LEU258 | 6.46 | 70.21% | THR215 | 5.55 | 95.16% | PRO89 | 2.59 | 62.70% | | | |
| | ARG309 | 8.51 | 69.49% | LYS243 | 6.31 | 93.09% | THR97 | 2.67 | 60.71% | | | |
| | CYS304 | 7.56 | 68.97% | LEU219 | 5.59 | 93.00% | | | | | | |
| | LEU300 | 7.52 | 67.95% | ALA246 | 6.34 | 91.71% | | | | | | |
| | SER254 | 6.42 | 65.25% | VAL253 | 6.41 | 90.88% | | | | | | |
| | ALA250 | 6.38 | 62.41% | SER254 | 6.42 | 88.47% | | | | | | |
| | SER248 | 6.36 | 61.75% | GLY257 | 6.45 | 86.60% | | | | | | |
| | LYS244 | 6.32 | 58.71% | ILE225 | 5.65 | 84.01% | | | | | | |
| | VAL296 | 7.48 | 58.45% | VAL214 | 5.54 | 83.60% | | | | | | |
| | TYR306 | 0.00 | 49.86% | ILE221 | 5.61 | 81.21% | | | | | | |
| | | | | PHE211 | 5.51 | 79.03% | | | | | | |
| | | | | TRP260 | 6.48 | 78.62% | | | | | | |
| | | | | LEU258 | 6.46 | 57.06% | | | | | | |
| | | | | PHE256 | 6.44 | 51.17% | | | | | | |
| | | | | ALA261 | 6.49 | 50.16% | | | | | | |

| | | | Residence time | | | Residence time | | | Residence time |
|---|---|---|---|---|---|---|---|---|---|
| DPPC | 65 | | 88.81% | 22 | | 85.25% | 59 | | 85.15% |
| Amino acid residue | LEU303 | 7.55 | 85.18% | ARG66 | 12.51 | 85.21% | TYR222 | 5.62 | 80.87% |
| | ARG309 | 8.51 | 76.62% | VAL60 | 1.58 | 85.21% | GLN226 | 5.66 | 65.76% |
| | VAL299 | 7.51 | 59.36% | MET56 | 1.54 | 84.93% | LEU219 | 5.59 | 57.52% |
| | TYR306 | 0.00 | 58.67% | PHE59 | 1.57 | 84.81% | | | |
| | | | | LEU74 | 2.44 | 84.65% | | | |
| | | | | PHE81 | 2.51 | 81.07% | | | |
| | | | | ILE78 | 2.48 | 80.02% | | | |
| | | | | SER63 | 12.48 | 73.90% | | | |
| | | | | LEU57 | 1.55 | 69.68% | | | |
| | | | | PHE71 | 2.41 | 64.01% | | | |
| | | | | ALA61 | 1.59 | 61.09% | | | |
| | | | | LEU82 | 2.52 | 59.72% | | | |
| | | | | ASP62 | 1.60 | 52.81% | | | |

Supplementary Table S5 (see below).

## H3Rapo

| | | | Residence time | | | Residence time | | | Residence time | | | Residence time | | | Residence time |
|---|---|---|---|---|---|---|---|---|---|---|---|---|---|---|---|
| DPPC | 184 | | 100.00% | 151 | | 98.25% | 133 | | 96.42% | 155 | | 88.47% | 128 | | 82.44% |
| Amino acid residue | TRP 281 | 7.40 | 100.00% | LEU109 | 3.27 | 98.74% | TRP33 | 1.31 | 73.40% | ARG27 | 0.00 | 79.35% | LEU258 | 6.53 | 80.12% |
| | GLU277 | 7.36 | 99.83% | LYS108 | 3.26 | 97.76% | LEU40 | 1.38 | 72.31% | PHE29 | 0.00 | 53.83% | ILE262 | 6.57 | 75.91% |
| | TYR274 | 7.33 | 99.27% | VAL112 | 3.3 | 95.42% | LEU37 | 1.35 | 59.57% | | | | PRO255 | 6.50 | 65.46% |
| | ALA285 | 7.44 | 98.93% | TYR167 | 4.57 | 94.57% | TYR274 | 7.33 | 58.91% | | | | TRP275 | 7.34 | 64.76% |
| | THR278 | 7.37 | 96.32% | VAL113 | 3.31 | 92.42% | | | | | | | CYS266 | 6.61 | 55.80% |
| | LEU44 | 1.42 | 95.96% | LEU116 | 3.34 | 92.05% | | | | | | | ALA254 | 6.49 | 53.68% |
| | LEU37 | 1.35 | 95.44% | ALA163 | 4.53 | 91.62% | | | | | | | LEU259 | 6.54 | 53.05% |
| | VAL95 | 2.65 | 95.14% | GLY105 | 3.23 | 89.95% | | | | | | | | | |
| | LEU40 | 1.38 | 90.32% | TRP160 | 4.5 | 82.08% | | | | | | | | | |
| | TYR91 | 2.61 | 90.18% | VAL159 | 4.49 | 82.04% | | | | | | | | | |
| | LEU282 | 7.41 | 88.36% | ARG104 | 3.22 | 78.15% | | | | | | | | | |
| | GLY98 | 2.68 | 86.11% | ALA170 | 4.6 | 66.44% | | | | | | | | | |
| | ALA288 | 7.47 | 74.47% | LEU82 | 2.52 | 66.34% | | | | | | | | | |
| | VAL289 | 7.48 | 73.43% | PHE164 | 4.54 | 61.74% | | | | | | | | | |
| | ASP273 | 7.32 | 70.32% | TYR176 | 45.38 | 61.73% | | | | | | | | | |
| | TRP33 | 1.31 | 65.56% | ILE171 | 4.61 | 61.59% | | | | | | | | | |
| | TYR94 | 2.64 | 63.54% | MET156 | 4.46 | 59.43% | | | | | | | | | |
| | LEU96 | 2.66 | 61.77% | LEU117 | 3.35 | 56.34% | | | | | | | | | |
| | LEU43 | 1.41 | 57.98% | | | | | | | | | | | | |
| | TRP284 | 7.43 | 53.40% | | | | | | | | | | | | |
| | THR97 | 2.67 | 52.68% | | | | | | | | | | | | |

Supplementary Table S6 (see below).

Tables S4, S5 and S6. Lipid-binding sites on the inactive-state (antagonist-bound), active-state (agonist-bound), and constitutive-state receptor (apo), respectively.

| | H3Ranta | | H3Rago | | H3Rapo | |
|---|---|---|---|---|---|---|
| | No .aa | % | No. aa | % | No. aa | % |
| non-polar, aliphatic | | | | | | |
| GLY | 3 | 2.88% | 1 | 1.69% | 2 | 4.00% |
| ALA | 0 | 0.00% | 5 | 8.47% | 5 | 10.00% |
| VAL | 9 | 8.65% | 7 | 11.86% | 4 | 8.00% |
| LEU | 26 | 25.00% | 10 | 16.95% | 14 | 28.00% |
| ILE | 5 | 4.81% | 6 | 10.17% | 2 | 4.00% |
| MET | 1 | 0.96% | 1 | 1.69% | 1 | 2.00% |
| polar, non-charged | | | | | | |
| SER | 5 | 4.81% | 4 | 6.78% | 0 | 0.00% |
| THR | 6 | 5.77% | 3 | 5.08% | 2 | 4.00% |
| CYS | 1 | 0.96% | 1 | 1.69% | 1 | 2.00% |
| PRO | 3 | 2.88% | 2 | 3.39% | 1 | 2.00% |
| ASN | 2 | 1.92% | 1 | 1.69% | 0 | 0.00% |
| GLN | 1 | 0.96% | 0 | 0.00% | 0 | 0.00% |
| aromatic | | | | | | |
| PHE | 11 | 10.58% | 5 | 8.47% | 1 | 2.00% |
| TYR | 6 | 5.77% | 3 | 5.08% | 6 | 12.00% |
| TRP | 4 | 3.85% | 1 | 1.69% | 6 | 12.00% |
| charged | | | | | | |
| LYS | 6 | 5.77% | 4 | 6.78% | 1 | 2.00% |
| ARG | 11 | 10.58% | 4 | 6.78% | 2 | 4.00% |
| HIS | 1 | 0.96% | 0 | 0.00% | 0 | 0.00% |
| ASP | 1 | 0.96% | 1 | 1.69% | 1 | 2.00% |
| GLU | 2 | 1.92% | 0 | 0.00% | 1 | 2.00% |
| | 104 | 100.00% | 59 | 100.00% | 50 | 100.00% |

Supplementary Table S7. Residue population in contact with the lipids grouped according to amino acid classes (non-polar aliphatic, uncharged polar, aromatic, and charged) for the antagonist, agonist and apo systems, respectively.

**Antagonist** complex

| DPPC | Motif | | | Region |
|---|---|---|---|---|
| 33 | $N^{45.57}$ | $Y^{5.37}$ | | ECL2, TM5 |
| 79 | $F^{0.00}$ | $^{7.33}YX_3TX_2W^{7.40}$ | | N-ter, TM7 |
| 110 | $^{3.41}FX_3LX_2Y^{3.48}$ | $L^{5.45}$ | | TM3, TM5 |
| 125 | $^{6.35}KX_3I^{6.39}$ | $^{7.55}LC^{7.56}$ | | TM6, TM7 |
| 161 | $F^{3.41}$ | $^{4.41}RX_2RKX_2L^{4.48}$ | | TM3, TM4 |

**Agonist** complex

| DPPC | Motif | | | Region |
|---|---|---|---|---|
| 47 | $^{5.55}TX_2NLX_2Y^{5.63}$ | $^{6.31}KX_2AKX_2AX_2V^{6.41}$ | | TM5, TM6 |

**Apo** receptor

| DPPC | Motif | | | Region |
|---|---|---|---|---|
| 151 | $^{3.23}GX_2KLX_2VVX_2L^{3.34}$ | $^{4.53}AX_3Y^{4.57}$ | | TM3, TM4 |
| 184 | $^{1.35}LX_2LX_3L^{1.42}$ | $^{2.61}YX_3V^{2.65}$ | $^{7.33}YX_2ETX_2WX_3A^{7.44}$ | TM1, TM2, TM7 |

Supplementary Table S8. Lipid binding H3R amino acid sequence motifs, resulting after selection of amino acid residues in contact with lipids that show residence times ≥ 90%.

| Anta | | Ago | | Apo | |
|---|---|---|---|---|---|
| MET41:TRP281 | 1.39:7.40 | MET41:TRP288 | 1.39:7.40 | MET41:TYR91 | 1.39:2.61 |
| | | | | MET41:TRP281 | 1.39:7.40 |
| MET41:TRP284 | 1.39:7.43 | | | MET41:TRP284 | 1.39:7.43 |
| | | | | ARG150:PRO291 | 1.50:7.50 |
| ASN69:ASP131 | 2.39:3.49 | | | MET56:PHE81 | 1.54:2.51 |
| ASN69:ARG132 | 2.39:3.50 | | | ASN69:ASP131 | 2.39:3.49 |
| | | | | ASN69:ARG132 | 2.39:3.50 |
| | | | | ASP80:PRO291 | 2.50:7.50 |
| ARG132:ASP131 | 3.50:3.49 | | | ASP114:TRP284 | 3.32:7.43 |
| ARG132:ASN224 | 3.50:5.64 | | | | |
| ARG132:ASP235 | 3.50:6.30 | | | | |
| PHE151:TRP253 | 5.51:6.48 | | | | |
| | | | | PHE207:TRP253 | 5.47:6.48 |
| MET260:TYR256 | 6.55:6.51 | MET267:PHE208 | 6.55:5.48 | TRP253:SER287 | 6.48:7.46 |
| | | MET267:TYR115 | 6.55:3.33 | MET260:TYR256 | 6.55:6.51 |
| MET260:PHE280 | 6.55:7.39 | MET267:TYR263 | 6.55:6.51 | | |
| | | TYR301:PHE308 | 7.53:8.50 | | |
| | | | | TYR294:PHE301 | 7.53:8.50 |
| | | | | TYR294:PHE305 | 7.53:8.54 |

Supplementary Table S9. Selected pairs of residues establishing contacts according to inter-residue distance criteria of 6 Å between COMs of side chains. Includes ionic locks Arg3.50-Asp3.49, and Arg3.50-Asp6.30.

## REFERENCES


(1) Amadei, A.; Linssen, A. B. M.; Berendsen, H. J. C. Essential Dynamics of Proteins. *Proteins: Structure, Function, and Bioinformatics* **1993**. https://doi.org/10.1002/prot.340170408.

(2) Grossfield A, Feller SE, P. MC. Convergence of Molecular Dynamics Simulations of Membrane Proteins. **2007**, *67* (1), 31–40. https://doi.org/10.1002/prot.21308.

(3) Faraldo-Gómez, J. D.; Forrest, L. R.; Baaden, M.; Bond, P. J.; Domene, C.; Patargias, G.; Cuthbertson, J.; Sansom, M. S. P. Conformational Sampling and Dynamics of Membrane Proteins from 10-Nanosecond Computer Simulations. *Proteins: Structure, Function and Genetics* **2004**. https://doi.org/10.1002/prot.20257.

(4) Tsoulos, I. G.; Stavrakoudis, A. Eucb: A C++ Program for Molecular Dynamics Trajectory Analysis. *Computer Physics Communications* **2011**. https://doi.org/10.1016/j.cpc.2010.11.032.

(5) Mezei, M. Simulaid: A Simulation Facilitator and Analysis Program. *Journal of Computational Chemistry* **2010**. https://doi.org/10.1002/jcc.21551.

(6) Mezei, M.; Filizola, M. TRAJELIX: A Computational Tool for the Geometric Characterization of Protein Helices during Molecular Dynamics Simulations. *Journal of Computer-Aided Molecular Design* **2006**. https://doi.org/10.1007/s10822-006-9039-1.

(7) Glykos, N. Software News and Updates. CARMA: A Molecular Dynamics Analysis Program. *Journal of computational chemistry* **2006**, *27*, 1765–1768. https://doi.org/10.1002/jcc.20482.

(8) Humphrey, W.; Dalke, A.; Schulten, K. VMD: Visual Molecular Dynamics. *Journal of Molecular Graphics* **1996**. https://doi.org/10.1016/0263-7855(96)00018-5.

(9) Schmidtke, P.; Le Guilloux, V.; Maupetit, J.; Tufféry, P. Fpocket: Online Tools for Protein Ensemble Pocket Detection and Tracking. *Nucleic Acids Research* **2010**. https://doi.org/10.1093/nar/gkq383.

(10) Hernandez, M.; Ghersi, D.; Sanchez, R. SITEHOUND-Web: A Server for Ligand Binding Site Identification in Protein Structures. *Nucleic Acids Research* **2009**. https://doi.org/10.1093/nar/gkp281.

(11) Chovancova, E.; Pavelka, A.; Benes, P.; Strnad, O.; Brezovsky, J.; Kozlikova, B.; Gora, A.; Sustr, V.; Klvana, M.; Medek, P.; Biedermannova, L.; Sochor, J.; Damborsky, J. CAVER 3.0: A Tool for the Analysis of Transport Pathways in Dynamic Protein Structures. *PLoS Computational Biology* **2012**. https://doi.org/10.1371/journal.pcbi.1002708.

(12) Rasmussen, S. G. F.; Choi, H.-J.; Fung, J. J.; Pardon, E.; Casarosa, P.; Chae, P. S.; Devree, B. T.; Rosenbaum, D. M.; Thian, F. S.; Kobilka, T. S.; Schnapp, A.; Konetzki, I.; Sunahara, R. K.; Gellman, S. H.; Pautsch, A.; Steyaert, J.; Weis, W. I.; Kobilka, B. K. Structure of a Nanobody-Stabilized Active State of the β(2) Adrenoceptor. *Nature* **2011**, *469* (7329), 175–80. https://doi.org/10.1038/nature09648.

(13) Shimamura, T.; Shiroishi, M.; Weyand, S.; Tsujimoto, H.; Winter, G.; Katritch, V.; Abagyan, R.; Cherezov, V.; Liu, W.; Han, G. W.; Kobayashi, T.; Stevens, R. C.; Iwata, S. Structure of the Human Histamine H1 Receptor Complex with Doxepin. *Nature* **2011**, *475* (7354), 65–70. https://doi.org/10.1038/nature10236.

(14) Klco, J. M.; Wiegand, C. B.; Narzinski, K.; Baranski, T. J. Essential Role for the Second Extracellular Loop in C5a Receptor Activation. *Nature Structural and Molecular Biology* **2005**. https://doi.org/10.1038/nsmb913.

(15) Peeters, M. C.; Van Westen, G. J. P.; Li, Q.; Ijzerman, A. P. *Importance of the Extracellular Loops in G Protein-Coupled Receptors for Ligand Recognition and Receptor Activation*; 2011. https://doi.org/10.1016/j.tips.2010.10.001.

(16) Rosenbaum, D. M.; Zhang, C.; Lyons, J. A.; Holl, R.; Aragao, D.; Arlow, D. H.; Rasmussen, S. G. F.; Choi, H.-J.; Devree, B. T.; Sunahara, R. K.; Chae, P. S.; Gellman, S. H.; Dror, R. O.; Shaw, D. E.; Weis, W. I.; Caffrey, M.; Gmeiner, P.; Kobilka, B. K. Structure and Function of an Irreversible

Agonist-β(2) Adrenoceptor Complex. *Nature* **2011**, *469* (7329), 236–40. https://doi.org/10.1038/nature09665.
(17) Rasmussen, S. G. F.; DeVree, B. T.; Zou, Y.; Kruse, A. C.; Chung, K. Y.; Kobilka, T. S.; Thian, F. S.; Chae, P. S.; Pardon, E.; Calinski, D.; Mathiesen, J. M.; Shah, S. T. A.; Lyons, J. A.; Caffrey, M.; Gellman, S. H.; Steyaert, J.; Skiniotis, G.; Weis, W. I.; Sunahara, R. K.; Kobilka, B. K. Crystal Structure of the B2 Adrenergic Receptor-Gs Protein Complex. *Nature* **2011**, *477* (7366), 549–55. https://doi.org/10.1038/nature10361.
(18) Hiller, C.; Weichert, D.; Gmeiner, P.; Hubner, H.; Zhang, C.; Manglik, A.; Kobilka, B. K.; Kruse, A. C. Covalent Agonists for Studying G Protein-Coupled Receptor Activation. *Proceedings of the National Academy of Sciences* **2014**. https://doi.org/10.1073/pnas.1410415111.
(19) Sounier, R.; Mas, C.; Steyaert, J.; Laeremans, T.; Manglik, A.; Huang, W.; Kobilka, B. K.; Déméné, H.; Granier, S. Propagation of Conformational Changes during μ-Opioid Receptor Activation. *Nature* **2015**, *524* (7565), 375–378. https://doi.org/10.1038/nature14680.
(20) Kiss, R.; Keserű, G. M. Structure-Based Discovery and Binding Site Analysis of Histamine Receptor Ligands. *Expert opinion on drug discovery* **2016**, *11* (12), 1165–1185. https://doi.org/10.1080/17460441.2016.1245288.
(21) Liu, W.; Chun, E.; Thompson, A. A.; Chubukov, P.; Xu, F.; Katritch, V.; Han, G. W.; Roth, C. B.; Heitman, L. H.; IJzerman, A. P.; Cherezov, V.; Stevens, R. C. Structural Basis for Allosteric Regulation of GPCRs by Sodium Ions. *Science* **2012**, *337* (6091), 232–236. https://doi.org/10.1126/SCIENCE.1219218.
(22) Yuan, S.; Palczewski, K.; Peng, Q.; Kolinski, M.; Vogel, H.; Filipek, S. The Mechanism of Ligand-Induced Activation or Inhibition of μ- And κ-Opioid Receptors. *Angewandte Chemie - International Edition* **2015**. https://doi.org/10.1002/anie.201501742.
(23) Kofuku, Y.; Ueda, T.; Okude, J.; Shiraishi, Y.; Kondo, K.; Maeda, M.; Tsujishita, H.; Shimada, I. Efficacy of the β 2-Adrenergic Receptor Is Determined by Conformational Equilibrium in the Transmembrane Region. *Nature Communications* **2012**. https://doi.org/10.1038/ncomms2046.
(24) Holst, B.; Rosenkilde, M. M.; Elling, C. E.; Frimurer, T. M.; Schwartz, T. W. MOLECULAR MECHANISM OF 7TM RECEPTOR ACTIVATION—A GLOBAL TOGGLE SWITCH MODEL. *Annual Review of Pharmacology and Toxicology* **2006**. https://doi.org/10.1146/annurev.pharmtox.46.120604.141218.
(25) Nygaard, R.; Frimurer, T. M.; Holst, B.; Rosenkilde, M. M.; Schwartz, T. W. *Ligand Binding and Micro-Switches in 7TM Receptor Structures*; 2009. https://doi.org/10.1016/j.tips.2009.02.006.
(26) Nieto-Alamilla, G.; Marquez-Gomez, R.; Garcia-Galvez, A.-M.; Morales-Figueroa, G.-E.; Arias-Montano, J.-A. The Histamine H3 Receptor: Structure, Pharmacology, and Function. *Molecular Pharmacology* **2016**. https://doi.org/10.1124/mol.116.104752.
(27) Dror, R. O.; Jensen, M. Ø.; Shaw, D. E. Elucidating Membrane Protein Function through Long-Timescale Molecular Dynamics Simulation. *Conf Proc IEEE Eng Med Biol Soc* **2009**, *2009*, 2340–2342. https://doi.org/10.1109/IEMBS.2009.5335057.
(28) Nygaard, R.; Zou, Y.; Dror, R. O.; Mildorf, T. J.; Arlow, D. H.; Manglik, A.; Pan, A. C.; Liu, C. W.; Fung, J. J.; Bokoch, M. P.; Thian, F. S.; Kobilka, T. S.; Shaw, D. E.; Mueller, L.; Prosser, R. S.; Kobilka, B. K. The Dynamic Process of B2-Adrenergic Receptor Activation. *Cell* **2013**. https://doi.org/10.1016/j.cell.2013.01.008.
